# A chemical and biological toolbox for Type Vd secretion: Characterization of the phospholipase A1 autotransporter FplA from *Fusobacterium nucleatum*

**DOI:** 10.1074/jbc.M117.819144

**Published:** 2017-10-11

**Authors:** Michael A. Casasanta, Christopher C. Yoo, Hans B. Smith, Alison J. Duncan, Kyla Cochrane, Ann C. Varano, Emma Allen-Vercoe, Daniel J. Slade

**Affiliations:** From the ‡ Department of Biochemistry, Virginia Polytechnic Institute and State University, Blacksburg, Virginia 24061,; § Canada's Michael Smith Genome Sciences Centre, British Columbia Cancer Agency, Vancouver, British Columbia V5Z 4S6, Canada,; the ¶ Department of Medical Genetics, University of British Columbia, Vancouver, British Columbia V6T 1Z4, Canada,; the ‖ Department of Biochemistry and Molecular Biology, Simon Fraser University, Vancouver, British Columbia V5A 1S6, Canada,; the ** Virginia Tech Carilion Research Institute, Roanoke, Virginia 24016, and; the ‡‡ Department of Molecular and Cellular Biology, University of Guelph, Guelph, Ontario N1G 2W1, Canada

**Keywords:** chemical biology, colorectal cancer, host-pathogen interaction, phospholipase A, protein secretion, Fusobacterium, autotransporter

## Abstract

*Fusobacterium nucleatum* is an oral pathogen that is linked to multiple human infections and colorectal cancer. Strikingly, *F. nucleatum* achieves virulence in the absence of large, multiprotein secretion systems (Types I, II, III, IV, and VI), which are widely used by Gram-negative bacteria for pathogenesis. By contrast, *F. nucleatum* strains contain genomic expansions of Type V secreted effectors (autotransporters) that are critical for host cell adherence, invasion, and biofilm formation. Here, we present the first characterization of an *F. nucleatum* Type Vd phospholipase class A1 autotransporter (strain ATCC 25586, gene FN1704) that we hereby rename *Fusobacterium* phospholipase autotransporter (FplA). Biochemical analysis of multiple *Fusobacterium* strains revealed that FplA is expressed as a full-length 85-kDa outer membrane–embedded protein or as a truncated phospholipase domain that remains associated with the outer membrane. Whereas the role of Type Vd secretion in bacteria remains unidentified, we show that FplA binds with high affinity to host phosphoinositide-signaling lipids, revealing a potential role for this enzyme in establishing an *F. nucleatum* intracellular niche. To further analyze the role of FplA, we developed an *fplA* gene knock-out strain, which will guide future *in vivo* studies to determine its potential role in *F. nucleatum* pathogenesis. In summary, using recombinant FplA constructs, we have identified a biochemical toolbox that includes lipid substrates for enzymatic assays, potent inhibitors, and chemical probes to detect, track, and characterize the role of Type Vd secreted phospholipases in Gram-negative bacteria.

## Introduction

*Fusobacterium nucleatum* is an emerging oral pathogen that is involved in periodontitis (1) and also readily disseminates, presumably through hematogenous spread (2, 3), to cause potentially fatal infections of the brain (4), liver (5), lungs (6), heart (7), appendix (8), and amniotic fluid, where it causes preterm birth (2, 9, 10). Recent studies have uncovered a correlation between colorectal cancer tumors and an overabundance of *F. nucleatum* present in diseased tissue (11–13). Subsequent studies confirmed a potential causative effect for *F. nucleatum* in tumor formation using an APC^min/−^ mouse model of accelerated CRC pathogenesis (14). In addition, human patients who had the highest detected levels of *F. nucleatum* within tumors had the lowest survival rate (15). Invasive *F. nucleatum* strains can enter into epithelial and endothelial cells (16, 17), which induces the secretion of proinflammatory cytokines that drive local inflammation, as seen in colorectal cancer (14). Previously characterized proteins involved in host cell binding and invasion include FadA (ATCC 25586, gene FN0264), a small helical adhesin that binds to E-cadherin and modulates prevalent colorectal cancer signaling pathways (18, 19); Fap2 (ATCC 25586, gene FN1449), a galactose-inhibitable Type Va secreted autotransporter adhesin that binds Gal-GalNAc sugars (3, 20–22); and RadD (ATCC 25586, gene 1526), an arginine-inhibitable Type Va autotransporter adhesin (20, 23). *F. nucleatum* also induces the production of human β-defensins 2 and 3 (hBD2 and hBD3), which are secreted, cationic antimicrobial peptides that act as chemoattractants to modulate adaptive immunity (24, 25).

*F. nucleatum* is unique in that it does not harbor large, multiprotein secretion systems (Types I–IV, VI, and IX) to establish infections and alter host signaling for survival (26). However, invasive strains of *F. nucleatum* contain an overabundance of uncharacterized proteins containing type II membrane occupation and recognition nexus (MORN2) domains and a genomic expansion of Type V secreted effectors known as autotransporters (17). Autotransporters are large outer membrane and secreted proteins that are divided into five classes (Types Va–Ve) based on their domain architecture and are critical proteins in host cell adherence, invasion, and biofilm formation (27–30). Autotransporter biogenesis and folding is driven by initial translocation through the SEC apparatus in the inner membrane, followed by the insertion of a C-terminal β-barrel domain in the outer membrane (30, 31). In a process that requires multiple chaperones (*e.g.* BAM complex), the large N-terminal passenger domain is present on the surface or cleaved and secreted after β-barrel translocation. The recent biochemical and structural characterization of the Type Vd autotransporter PlpD from *Pseudomonas aeruginosa* revealed a secreted N-terminal patatin-like protein (PFAM: PF01734) with an α-β hydrolase fold containing a catalytic dyad (Ser and Asp) conferring phospholipase A1 activity (EC 3.1.1.32) through the hydrolysis of glycerophospholipid moieties at the *sn*-1 position to release a fatty acid (32, 33). In addition, PlpD contains a 16-strand C-terminal β-barrel domain of the bacterial surface antigen family (PFAM: PF01103) for outer membrane anchorage, and a (polypeptide transport–associated; POTRA) domain potentially involved in protein folding and export of the phospholipase domain to the surface. The PlpD secreted phospholipase domain was able to disrupt liposomes and was also shown to bind the phosphoinositide class of human intracellular signaling lipids (34). Our analysis revealed that most *F. nucleatum* genomes each contain one gene (in strain ATCC 25586, gene FN1704, UniProtKB-Q8R6F6; herein renamed *fplA*) encoding for a previously uncharacterized ∼85-kDa Type Vd autotransporter that is homologous to PlpD. Bioinformatic analysis of *F. nucleatum* strains reveals that most strains also contain a single gene encoding for an additional small patatin domain–containing protein (∼32 kDa) (FN0508, UniProtKB-Q8R6A1) that is not a Type Vd autotransporter and does not contain a predicted signal sequence for export from the bacterial cytoplasm.

Whereas the role of Type Vd secreted phospholipases has not been determined, bacterial phospholipases play critical roles in the virulence of intracellular bacteria by promoting phagosome survival or by aiding in vacuole lysis to achieve liberation into the cytoplasm and subversion of host lysosomal induced death (35, 36). Bacterial pathogens, including *Helicobacter*, *Listeria*, *Salmonella*, *Shigella*, *Pseudomonas*, and *Legionella*, rely on phospholipases for virulence, survival, and some for intercellular spread (36). PldA1 from *Helicobacter pylori* is a phospholipase that is involved in growth at low pH (37), colonization of the gastric mucosa (38), and hemolytic activity (38). *Listeria monocytogenes* secretes two phospholipase C proteins (PI-PLC and PC-PLC) that are critical for late time point evasion of autophagy and establishment of an intracellular niche (39).

Here, we present studies that probe the molecular mechanisms of the Type Vd secreted autotransporter phospholipase FplA using a diverse set of chemical and biological tools. These experiments have strengthened our understanding of the Type Vd secretion and will aid in determining the role of FplA in *F. nucleatum* pathogenesis.

## Results

### FN1704 encodes for a Type Vd phospholipase autotransporter

**Figure 1.**
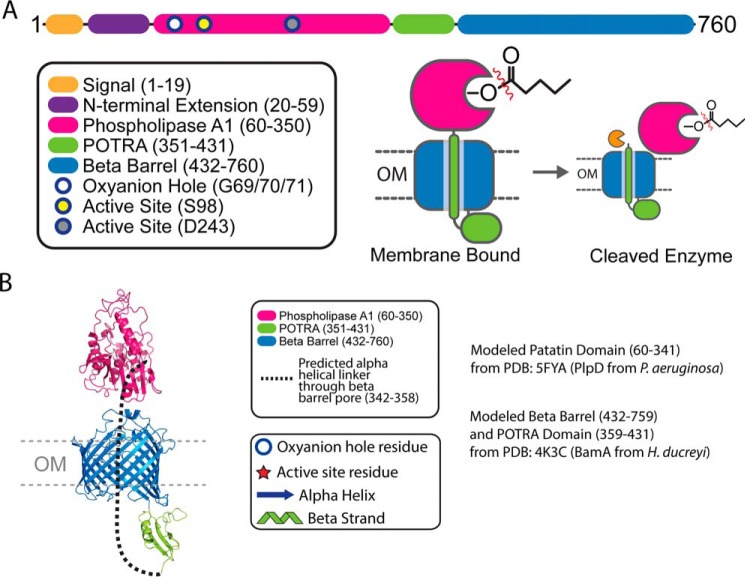
**Fig. 1 FplA is a Type Vd autotransporter phospholipase from *F. nucleatum*.** *A*, *schematic representation* of FplA domains and their location in the periplasm, outer membrane, and surface exposure of the PLA_1_ domain. Experimental data show FplA to be cleaved in a select set of *F. nucleatum* species, but the phospholipase domain remains associated with the bacterium. *B*, structure prediction of FplA domains: modeled patatin domain (residues 60–341) from PDB entry 5FYA (PlpD from *P. aeruginosa*); modeled β barrel (residues 432–759) and POTRA domain (residues 359–431) from PDB entry 4K3C (BamA from *H. ducreyi*).

**Figure 2.**
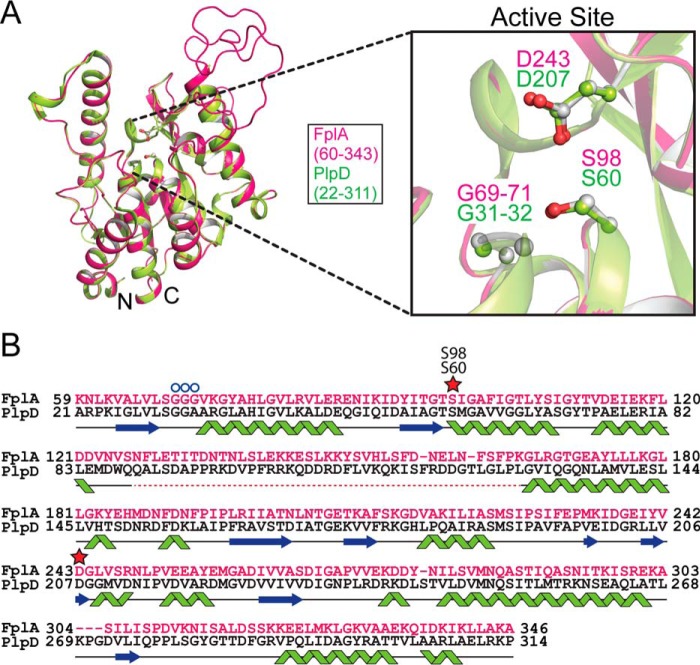
**A predicted FplA structure is homologous to PlpD.** *A*, alignment of a predicted FplA PLA1 domain structure with the crystal structure (PDB entry 5FQU) of the homologous phospholipase A_1_ enzyme PlpD from *P. aeruginosa*, with a magnified view of the catalytic dyad (Ser-98, Asp-243) and oxyanion hole (Gly-69, Gly-70, Gly-71). *B*, alignment of amino acids from PlpD (*black*) and FplA (*pink*) from the predicted structures of the PLA1 domains. The *dashed red line* indicates that this region was not predicted in the structure.

**Figure 3.**
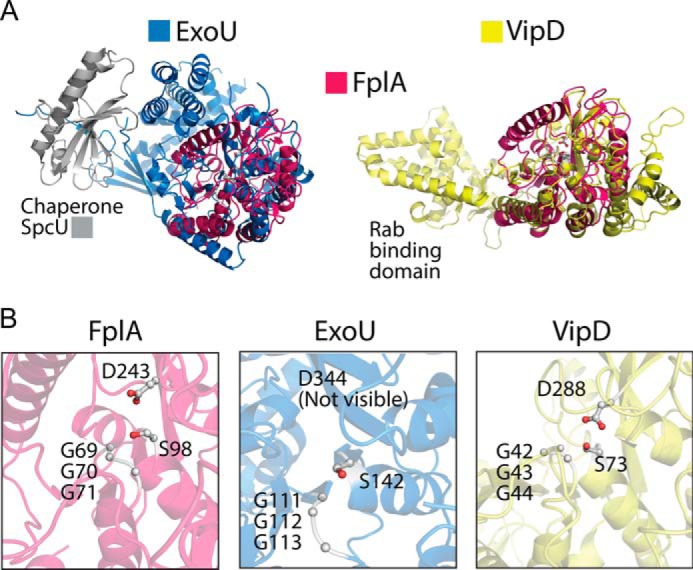
**Alignment of predicted FplA patatin domain structure with characterized phospholipase virulence factors.** *A*, predicted FplA structure (residues 60–431) aligned with ExoU (*P. aeruginosa*) (PDB entry 4AKX) and VipD (*L. pneumophila*) (PDB entry 4AKF). *B*, *zoomed in* view of active sites after alignment showing similar architectures and residue placement of the catalytic dyad (Ser and Asp) and oxyanion hole (Gly, Gly, and Gly).

*Fusobacterium* phospholipase autotransporter (FplA, UniProtKB-Q8R6F6) was identified as the gene previously labeled FN1704 in *F. nucleatum* ATCC 25586. Domain identification was carried out using SignalP version 4.1 to identify a signal sequence (residues 1–19), and the SWISS-MODEL (40) structure prediction server identified a patatin domain responsible for phospholipase activity (residues 60–350), a POTRA domain common in protein–protein interactions (residues 351–431), and a C-terminal β-barrel domain (residues 431–760) to insert FplA in the outer membrane (Fig. 1). In addition, we identified a unique 40-amino acid N-terminal extension (residues 20–59) that plays a role in the catalytic efficiency of the enzyme, probably by being critical for proper protein folding and position of the active site residues, and not substrate binding. Structure prediction of this enzyme revealed that the N-terminal patatin domain is highly similar to PlpD from *Pseudomonas aeruginosa* (PDB^2^ entry 5FQU), and an alignment shows an overall fold in residues 60–343 (32% identity corresponding to PlpD residues 22–311), which align well, with a highly conserved active site containing a catalytic dyad (Ser-98 and Asp-243) and an oxyanion hole (Gly-69/70/71) (Fig. 2). In addition, the next closest structural homologs of the FplA catalytic domain (residues 60–343) are predicted to be the non-autotransporter phospholipase A enzymes ExoU (Type III secreted) from *P. aeruginosa* (19.0% identity to residues 102–472; PDB entries 4AKX and 3TU3) and VipD (Type IV secreted) from *Legionella pneumophila* (17.3% identity to residues 33–411, PDB entry 4AKF) (Fig. 3).

### Characterization of fluorogenic substrates to probe the phospholipase A_1_ (PLA_1_) activity of FplA

**Figure 4.**
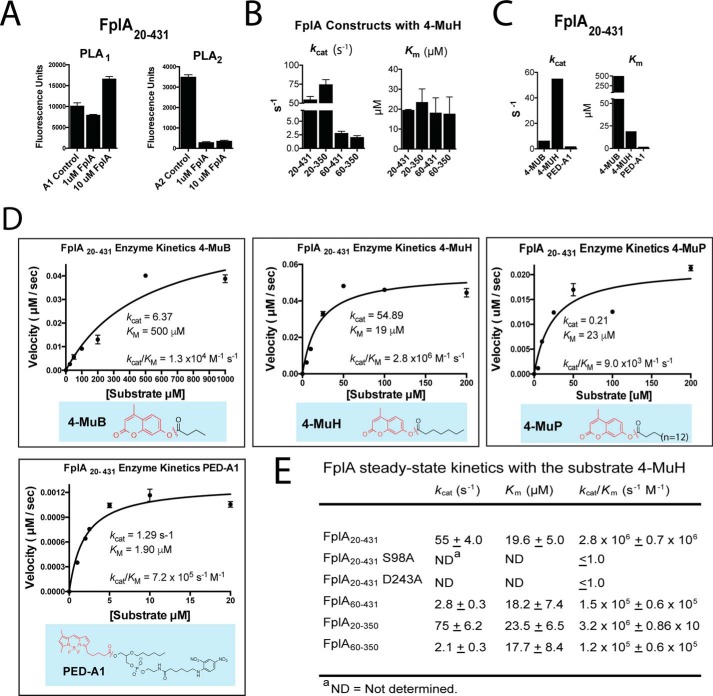
**Characterization of FplA lipase activity with multiple fluorescent substrates.** *A*, enzymatic assays show that FplA is a PLA_1_-specific enzyme with no PLA2 activity. *B*, steady-state kinetics of multiple FplA constructs with 4-MuH. *C*, FplA(20–431) *K*_cat_ and *K_m_* values vary greatly across substrates with various acyl chain lengths. *D* and *E*, characterization of FplA enzyme kinetics.

Multiple FplA constructs were cloned from the *F. nucleatum* 25586 genome and expressed in *E. coli*, including variations that lack a signal sequence for cytoplasmic expression (residues 20–431, 20–350, 60–431, and 60–350), and a full-length version in which we replaced the native signal sequence with an *E. coli* OmpA signal for more robust expression and surface presentation (OmpA(1–27)-FplA(20–760)). Constructs were tested for their phospholipase activity using substrates specific for either A1 or A2 class enzymes, as the homolog PlpD from *P. aeruginosa* showed specific A1 activity. We showed that FplA has only PLA_1_ activity (Fig. 4*A*) using the PLA_1_-specific substrate PED-A1 and further demonstrated that the general lipase substrates 4-methylumbelliferyl butyrate (4-MuB) and 4-methylumbelliferyl heptanoate (4-MuH) are robust tools for studies of FplA (Fig. 4, *B–E*). In addition, we determined that this enzyme is not dependent on calcium for activity (supplemental Fig. S1*A*) and that it is most active at pH 8.5 (supplemental Fig. S1*B*). The first full Michaelis–Menten kinetics for a Type Vd autotransporter were performed on each FplA construct using 4-MuH as a substrate and indicated that amino acids 20–350, incorporating the N-terminal extension and catalytic PLA_1_ domain, show the most robust catalytic efficiency (*k*_cat_/*K_m_* = 3.2 × 10^6^ s^−1^ m^−1^) (Fig. 4*E* and supplemental Fig. S1 (*C* and *D*)). Upon removal of the N-terminal extension, constructs had lower substrate turnover rates (*k*_cat_), but the relative binding affinities (*K_m_*) for 4-MuH were unchanged. We also show that tighter binding was seen with the substrate that most closely mimics a phospholipid (PED-A1, *K_m_* = 1.90 μm), and of the single acyl chain substrates, 4-MuH (7-carbon acyl chain) resulted in significantly tighter binding (*K_m_* = 19 μm) than with the 4-carbon acyl chain substrate 4-MuB (*K_m_* = 500 μm) (Fig. 4*C*). Mutation of the active site serine (S98A) and aspartate (D243A) residues that make up the catalytic dyad resulted in no detectable enzymatic activity (Fig. 4*E*). In addition, the glycine-rich stretch that constitutes the oxyanion hole (Gly-69/70/71) was analyzed, but Gly → Ala single mutations or multiple glycine changes (G69A/G70A/G71A) rendered the proteins insoluble^3^ and therefore could not be used for enzymatic analysis.

### Identification of FplA inhibitors and chemical probes for in vitro enzyme characterization

**Figure 5.**
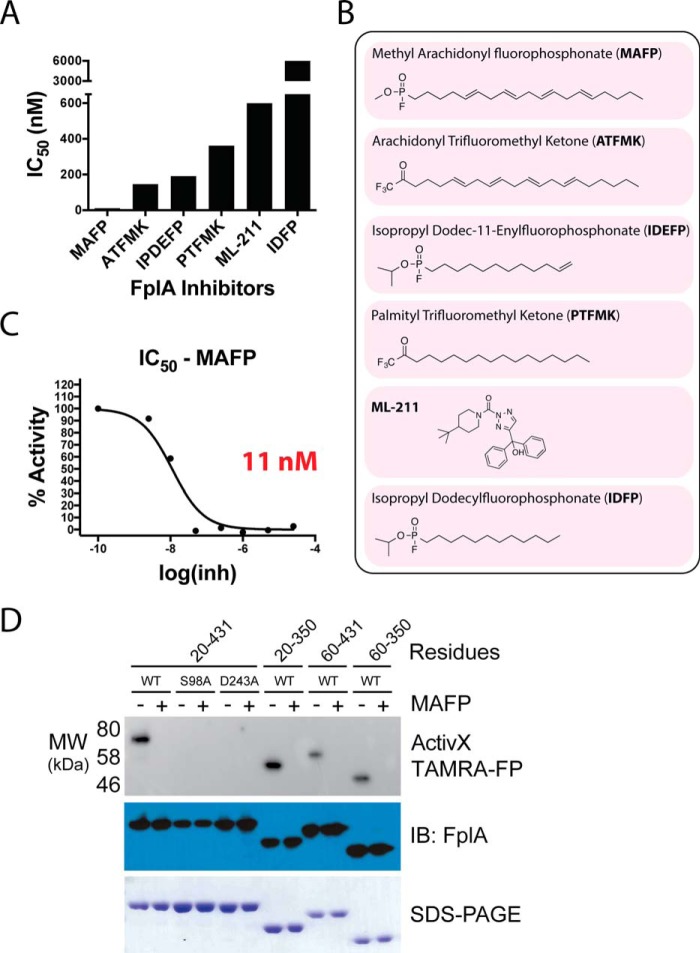
**Characterization of FplA inhibitors.** *A*, IC_50_ assays showing varying degrees of inhibition toward FplA by inhibitors previously shown to inhibit a variety of lipases. *B*, structure and names of tested inhibitors. *C*, IC_50_ plot of MAFP, the most potent (11 nm) FplA inhibitor characterized. *D*, analysis of the active site of FplA shows that the active site serine (Ser-98) reacts with ActivX TAMRA-FP probe but does not bind in the presence of the competitive inhibitor MAFP. S98A and D243A mutants will not bind the serine active site probe. Western blotting (*IB*) and SDS-polyacrylamide gels stained with Coomassie Blue serve as load controls for all constructs.

We present the first characterization of inhibitors for Type Vd autotransporter phospholipases. We show that the classic calcium-dependent PLA_2_ inhibitor methylarachidonyl fluorophosphonate (MAFP) is the most potent for FplA with an IC_50_ of 11 nm (41). Additional potent inhibitors contained a trifluoromethyl ketone headgroup (arachidonyl trifluoromethyl ketone; ATFMK), which also covalently binds to active-site serines within enzymes or an enylfluorophosphonate group (Fig. 5, *A–C*). We observed that isopropyl dodec-11-enylfluorophosphonate (IDEFP) is a much more potent inhibitor than isopropyl dodecylfluorophosphonate (IDFP); these compounds differ by only a double bond at the end of the IDEFP acyl chain. In addition, MAFP is the most potent inhibitor, and the arachidonyl portion of the molecule contains four double bonds, making it and ATFMK the most unsaturated substrates of the inhibitors tested. We therefore hypothesize that FplA binds and docks unsaturated acyl chain substrates and inhibitors with much higher affinity than saturated acyl chains, potentially because of angular changes within the molecule at double bonds. Additional inhibitors were tested that showed no significant activity against FplA (IC_50_ > 100 μm), and their analysis as well as IC_50_ plots for all inhibitors are presented in supplemental Fig. S2.

An activity-based protein profiling probe (ActivX TAMRA-FP) that labels active site serines in serine hydrolases was used to label purified FplA constructs (Fig. 5*D*) (42, 43). ActivX TAMRA-FP labeled active FplA but did not bind to the S98A or D243A mutants. We propose that in the absence of Asp-243, which stabilizes substrate, the probe does not properly interact with Ser-98 to initiate covalent labeling. In addition, in the presence of the competitive inhibitor MAFP, the ActivX TAMRA-FP probe is unable to bind to FplA due to competitive inhibition (Fig. 5*D*). We further demonstrated that load controlling is even by transferring the probe-bound proteins to PVDF and immunoblotting using a custom FplA(20–431) antibody.

### Expression of full-length FplA on the surface of E. coli

**Figure 6.**
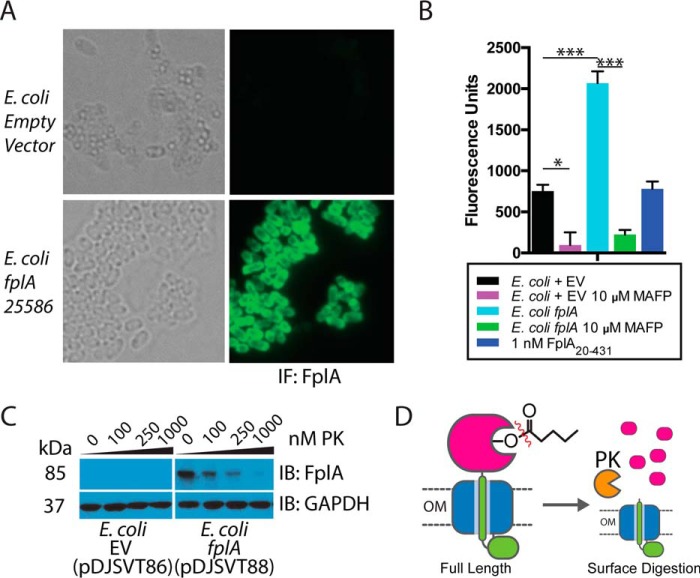
**Expression of full-length FplA in *E. coli* and functional analysis.** *A*, an OmpA(1–27) signal sequence allows for robust expression of FplA(20–760) on the surface of *E. coli* as seen by fluorescence microscopy with an anti-FplA antibody. *B*, enzymatic activity of FplA when live *E. coli* is added to reactions containing 4-MuH as a fluorescent substrate. *C*, PK, a cell-impenetrable nonspecific protease, is able to digest surface-exposed FplA, but not the cytoplasmic protein GAPDH. *D*, schematic of PK cleavage of full-length FplA from the surface of *E. coli. EV*, empty vector. Statistical analysis was performed using a multiple-comparison analysis by one-way analysis of variance. *, *p* ≤ 0.05; ***, *p* ≤ 0.0005. *Error bars*, S.D. *IB*, immunoblotting.

We created a FplA construct from *F. nucleatum* 25586 for recombinant expression in *E. coli* that removed the native signal sequence (residues 1–19) and replaced it with the signal sequence from *E. coli* OmpA (residues 1–27). This resulted in more robust expression of FplA on the surface of *E. coli* when compared with using a native signal sequence, which may not be recognized as efficiently by the *E. coli* Sec machinery (native signal sequence data not shown). We demonstrated that FplA can be efficiently exported through the Sec apparatus and assembled in the outer membrane, and the PLA_1_ domain of FplA is present and functional on the surface of *E. coli*. In Fig. 6*A*, we show that full-length FplA was detected on the surface of *E. coli* by fluorescence microscopy. FplA on the surface was active, as the addition of whole live bacteria to a reaction containing the fluorogenic substrate 4-MuH resulted in cleavage of the lipid substrate and a subsequent increase in fluorescence, which was inhibited by the addition of MAFP (Fig. 6*B*). To further prove that full-length FplA is expressed on the surface of *E. coli*, we confirm that treatment with the nonspecific and cell-impermeable protease, Proteinase K (PK), cleaves FplA from the surface but does not cleave the cytoplasmic control GAPDH (Fig. 6, *C* and *D*).

Attempts to detect FplA on the surface of *F. nucleatum* 23726 and *F. nucleatum* 25586 by fluorescence microscopy were unsuccessful, which we attribute to the low abundance of this protein as indicated by the need to use large cell quantities to see the protein via Western blot. It is possible that this is because FplA is such a potent phospholipase that high expression of the enzyme could be detrimental to *F. nucleatum*, as it could result in self-lysis and cell death. Additionally, we were unable to detect enzymatic activity by placing wild-type *F. nucleatum* 23726 directly in a mixture of 4-MuH substrate (results not shown). Neither of these negative results for activity are surprising, considering the low amount of FplA present; such a lack of activity at the surface is not uncommon for other outer membrane phospholipases in Gram-negative bacteria. For example, outer membrane phospholipase A from *E. coli* displays no activity in the absence of outer membrane destabilization compounds, such as polymyxin B (44).

### Creation of an fplA deletion strain in F. nucleatum 23726

**Figure 7.**
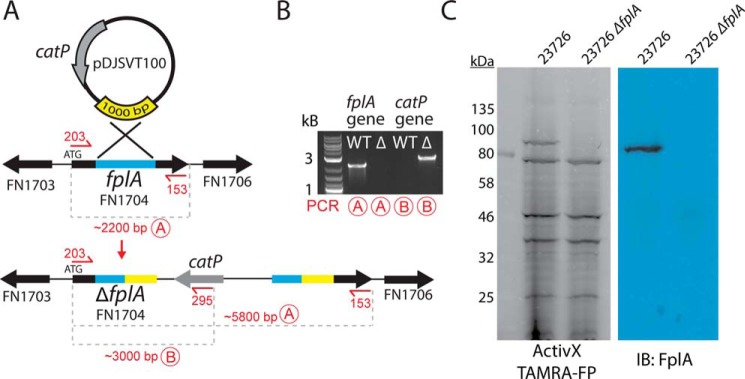
**Creation of an *F. nucleatum* 23726 Δ*fplA*.** *A*, pDJSVT100 is a single-crossover integration plasmid for disruption of the *fplA* gene. Primers are *labeled* in *red* for PCRs *A* and *B* to confirm plasmid integration and gene knock-out. *B*, PCR confirmation of the *F. nucleatum* 23726 Δ*fplA* strain. *C*, analysis of FplA protein (85.6 kDa) in WT and Δ*fplA* by fluorescent chemical probe (ActivX TAMRA-FP) to label all active site serine enzymes in the bacteria (also serves as a load control), followed by transfer to PVDF for Western blot analysis by probing with an anti-FplA antibody.

Genetic manipulation of *Fusobacterium* spp. is technically challenging, and of the seven strains used for analysis in this paper, only *F. nucleatum* 23726 and 10953 have been successfully mutated by gene deletion (45). A single homologous crossover plasmid (pDJSVT100; supplemental Table S2) that we developed from a *Clostridium* shuttle vector (46) using a recombination method previously established for *F. nucleatum* (45) was used to create a Δ*fplA* strain (gene HMPREF0397_1968) (strain DJSVT01; supplemental Table S1) marked with chloramphenicol resistance (Fig. 7, *A* and *B*). We verified by PCR that the *fplA* gene was disrupted by the chromosomally inserted plasmid and further showed expression of the protein had been abolished by a fluorescent probe and Western blots probed with an anti-FplA antibody (Fig. 7*C*). As phospholipases have been shown to play a role in bacterial membrane maintenance, we tested *F. nucleatum* 23726 Δ*fplA* for changes in growth rates and cell size and found that when compared with wild-type *F. nucleatum* 23726, there were no changes in these physical parameters when grown under standard laboratory conditions (supplemental Fig. S3).

### F. nucleatum strains express FplA as a full-length outer membrane protein or as a cleaved phospholipase domain that remains associated with the bacterial surface

**Figure 8.**
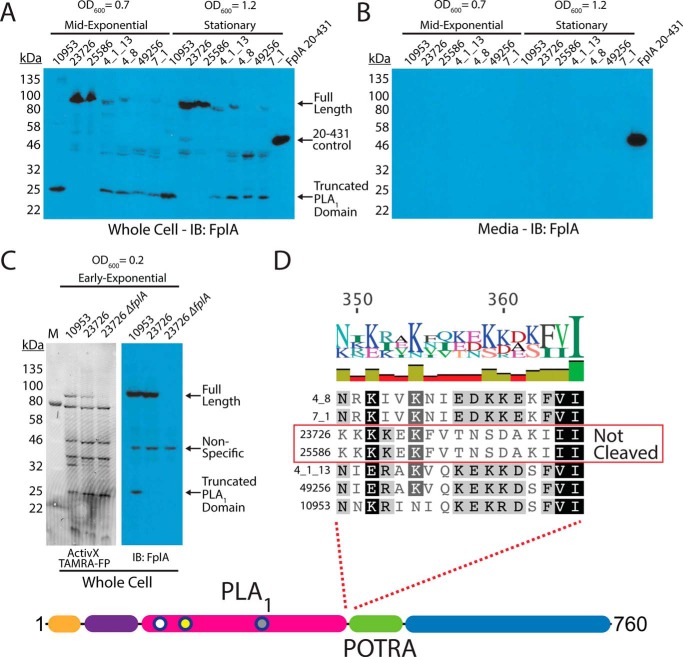
**Western blot analysis of FplA in multiple *Fusobacterium* strains.** *A*, initial characterization of FplA expression and protein size in mid-exponential phase (*A*_600_ = 0.7) and stationary phase (*A*_600_ = 1.2) shows that several strains produce a truncated form of FpA that consists of the PLA_1_ domain to which the FplA antibody was raised. *B*, Western blotting of medium from *Fusobacterium* growths shows that while truncated, FplA is not released into the medium and remains associated with the bacteria. *C*, analysis of FplA expression during early exponential phase growth (*A*_600_ = 0.2) reveals that strain 10953, which is cleaved in mid-exponential and stationary phase, is still in full-length state with a portion beginning to be cleaved. *D*, sequence alignment reveals that all FplA sequences from cleaved strains contain a highly charged motif at the PLA_1_/POTRA hinge region as a potential site for an unidentified protease, with the exception being the non-cleaved FplA proteins from 23726 and 25586, which contain a drastically different neutral motif. *IB*, immunoblotting.

Our initial results showed that FplA from *F. nucleatum* 23726 was expressed as a full-length 85-kDa protein, with no apparent release of the PLA_1_ domain from the β-barrel domain. Because PlpD from *P. aeruginosa* is a Type Vd autotransporter that releases the PLA_1_ domain into the medium, we sought to determine whether FplA from seven different *F. nucleatum* strains had different expression patterns or actual physical differences in the size or location of expressed and/or secreted domains. Various FplA proteins were expressed as either full-length 85-kDa proteins (strains 23726 and 25586) or as truncated phospholipase domains (FplA antibody developed against the PLA_1_ and POTRA domains) around 25–30 kDa for strains 10953, 4_8, 4_1_13, 49256, and 7_1 when expressed in either mid-exponential (*A*_600_ = 0.7) or stationary phase (*A*_600_ = 1.2) (Fig. 8*A*). Interestingly, we could not detect any secreted FplA in the spent culture medium, as was previously seen for PlpD from *P. aeruginosa* (Fig. 8*B*). We then tested for the presence of full-length FplA in 10953 (cleaved) and 23726 (uncleaved) in early exponential growth (*A*_600_ = 0.2) and found that we could detect full-length and truncated FplA from 10953, indicating that upon increases in bacterial cell density, FplA is cleaved from the surface by an unknown protein and mechanism (Fig. 8*C*). It is possible that FplA cleavage from the surface results in an active PLA_1_ domain that remains associated with the surface until released by undetermined host factors (pH, molecular cues, etc.) while colonizing specific regions of the human body.

Whereas the FplA amino acid sequences from the seven tested strains are highly similar (>95% identity), we identified two regions in *F. nucleatum* 23726 and *F. nucleatum* 25586 at the intersection of the end of the N-terminal extension and just before the end of the PLA_1_ domain, which could correspond to potential protease-processing sites (Fig. 8*D* and supplemental Fig. S4). The suspected cleavage site in *F. nucleatum* 23726 and *F. nucleatum* 25586 flanking the PLA_1_ domain is switched from a highly charged motif (consensus sequence: KNIEDKKEKF) to a more neutral motif (consensus sequence: KFVTNSDAKI) that could be more protease-resistant, resulting in retention of the full-length protein. In addition, to arrive at the 25-kDa product seen in five strains, a second cleavage event could occur at the end of the N-terminal extension, as strains 23726 and 25586 differ in this region by substitution of an alanine for charged and polar residues (supplemental Fig. S4).

### FplA binds phosphoinositide-signaling lipids

**Figure 9.**
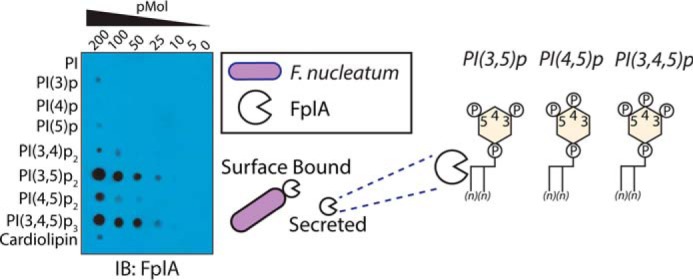
**FplA binds with high affinity to phosphoinositide-signaling lipids that are critical for multiple cellular processes in a human host.**

We tested FplA for binding to lipids found in human cells and found that it preferentially binds to human phosphoinositides, as was previously seen when characterizing the homologous enzyme PlpD from *P. aeruginosa* (33) (supplemental Fig. S5). Upon incubation with a more diverse and freshly prepared library of PIs, FplA was found to preferentially bind to PI(4,5)P_2_ and, with even stronger affinity, to PI(3,5)P_2_ and PI(3,4,5)P_3_ lipids (Fig. 9). This is consistent with structurally homologous enzymes binding PIs and implicates a role for this enzyme in an intracellular environment.

## Discussion

Fusobacterium are unique among Gram-negative bacteria in that the most recent and complete tree of life depicts a genetic lineage of *Fusobacterium* closer to high GC content Gram-positive bacteria (*e.g. Actinobacteria*) and Gram-negative *Bacteroidetes*, in which *Bacteroides fragilis* has multiple FplA homologs (47, 48). In addition, several *F. nucleatum* genes involved in metabolism are evolutionarily similar to those of Gram-positive *Clostridium* spp. (49). This unique combination of both Gram-positive and Gram-negative features could be an evolutionary clue as to why *Fusobacterium* lacks most Gram-negative specific secretion systems other than Type V.

Seminal studies by multiple groups have shown a repertoire of both small (FadA, ∼15 kDa) and large (Fap2, >300 kDa, Type Va secreted) *F. nucleatum* adhesins that are critical for host cell binding, invasion, and inflammation (3, 18). We set out to probe the role of a potential Type Vd virulence factor that we predicted to have phospholipase activity. We characterized the gene FN1704, which we have renamed *fplA* for *Fusobacterium* phospholipase autotransporter (FplA). Our *in vitro* studies were focused on identifying tools and methods to characterize Type Vd secreted autotransporters to determine their role in virulence in a diverse set of Gram-negative bacteria; many such autotransporters have been identified in intracellular pathogens (32). We created an *F. nucleatum* 23726 Δ*fplA* strain, which will allow us to next probe the role of this enzyme through the first *in vivo* studies of Type Vd autotransporter phospholipases in infection. Our analyses indicate that deletion of the *fplA* gene from *F. nucleatum* does not alter growth or cell size and shape under laboratory growth conditions, adding to our hypothesis that FplA is a potential virulence factor and not a bacterial maintenance protein.

The determination that different *F. nucleatum* strains express mature FplA proteins of varying molecular weights was a surprising result that made us question which form of the enzyme may be involved during specific *in vivo* niches within the human host. Because of the well-known genetic intractability of most *Fusobacterium* spp., we have not been able to delete copies of *fplA* in strains that we predicted to have a truncated yet surface-associated version of the protein. The development of more robust genetic systems for *Fusobacterium* has the potential to open doors to fill a critical knowledge gap in the role of Type Vd secretion in a variety of clinically isolated *F. nucleatum* strains.

Our initial results showed that FplA does not bind with high affinity to PA, PC, and PE, but these results do not rule out potential cleavage of these lipids in experiments that better simulate an environment found in infection. FplA could be involved in cleaving lipids in a mucous-rich environment found in the human oral cavity or gut. To add to the role of bacterial phospholipases cleaving lipids found in structural membranes, ExoU plays a major role in *P. aeruginosa* entry into the bloodstream upon leaving the lungs (50), and strains lacking ExoU are cleared more efficiently in mouse models of pneumonia (51, 52). Because cases of *Fusobacterium* bacteremia are frequently documented (*F. nucleatum* comprises 61% of cases) (53), and a wide array of bodily locations have been reported for *F. nucleatum* infections (brain (54), liver (55), lungs (6), and heart (56)), it will be critical to use our newly created *fplA* deletion strain to test the role of this enzyme in the previously established hematogenous spread (3).

As there are an impressive number of phosphoinositide-modulating enzymes secreted by bacteria to alter host signaling and induce colonization, it will be important to develop a robust set of chemical and molecular tools to determine the role of Type Vd surface-bound or secreted PLA_1_ enzymes in bacterial virulence. In summary, we have used chemical and biochemical tools to show that FplA is the lone Type Vd PLA_1_ enzyme found in *F. nucleatum* and is a potential virulence factor that modulates host–pathogen interactions.

## Experimental procedures

### Bacterial strains, growth conditions, and plasmids

Unless otherwise indicated, *E. coli* strains were grown in LB at 37 °C aerobically, and *F. nucleatum* strains were grown in CBHK (Columbia Broth, hemin (5 μg/ml), and menadione (0.5 μg/ml)) at 37 °C in an anaerobic chamber (90% N_2_, 5% CO_2_, 5% H_2_). Where appropriate, antibiotics were added at the indicated concentrations: carbenicillin, 100 μg/ml; thiamphenicol, 5 μg/ml (CBHK plates) or 2.5 μg/ml (CBHK liquid). For taxonomy verification of *Fusobacterium*, PCR amplification of a 1502-bp region of the 16S rRNA gene sequence was carried out using the universal primers U8F and U1510R (supplemental Table S3) as described previously (57). Sanger sequence analysis was carried out at the Advanced Analysis Center at the University of Guelph. Obtained DNA sequences were compared with the GenBank^TM^ database (NCBI) using BLASTn.

### Bioinformatic analysis of fplA in multiple Fusobacterium strains

The genome sequence of *F. nucleatum* strain ATCC 25586 (GenBank^TM^ accession number NC_003454.1) was used to predict all open reading frames using the Prodigal Bacterial Gene Prediction Server (58). An open reading frame encoding for a 760-amino acid protein was identified using an HMMER model built from a seed alignment of the PFAM (EMBL-EBI website) patatin family (PF01734) and the stand alone HMMER version 3.1 software package (59). The identified gene contained an N-terminal patatin domain conferring phospholipase activity and a C-terminal bacterial surface antigen domain (PFAM: PF01103) that encodes for an outer membrane β-barrel domain. Cross-referencing revealed that this gene is FN1704 in *F. nucleatum* ATCC 25586, which was incorrectly predicted to be a serine protease in both the KEGG and Uniprot databases. The same method was used to search multiple *Fusobacterium* genomes, resulting in the identification of only one protein with this structure in each strain. A PSI-BLAST search using FplA returned a close match to the *P. aeruginosa* protein PlpD, which was previously characterized as a class A_1_ phospholipase and labeled as the first in a new class of Type Vd autotransporters (32, 33). Alignment of FplA proteins from seven strains of *Fusobacterium* shown in supplemental Fig. S4 was performed using Geneious version 9.0.2 (60).

### Structure prediction to identify domain boundaries and catalytic residues in FplA

Structure prediction was performed using the FplA sequence from *F. nucleatum* strain 25586 and the SWISS-MODEL Workspace (61). Results showed a close match of the N-terminal phospholipase domain to PlpD from *P. aeruginosa* (PDB entry 5FYA) and the C-terminal POTRA and β-barrel domains to BamA from *Haemophilus ducreyi* (PDB entry 4K3C) (Figs. 1 and 2). A composite predicted structure was assembled using the predicted phospholipase, POTRA, and β-barrel domain, which has the phospholipase domain exposed on the surface of the bacteria, which we confirmed biochemically as a recombinant protein in *E. coli* and a native protein in *F. nucleatum*. In addition, the modeled FplA phospholipase domain was aligned with ExoU (PDB entry 4AKX) and VipD (PDB entry 4AKF) (Fig. 3). Active site residues in FplA were identified as Ser-98 and Asp-243, and these were verified by multiple enzymatic and chemical biology methods presented in Figs. 4 and 5. In close proximity to the active site is the oxyanion hole composed of three consecutive glycine residues (Gly-69, -70, and -71). Graphical representations and alignments of all predicted structures were created using the PyMOL Molecular Graphics System, version 1.7.3 (Schrödinger, LLC, New York).

### Cloning of FplA constructs for expression in E. coli

All primers were ordered from IDT DNA, and all plasmids and bacterial strains either used in or created for these studies are described in supplemental Table S1 (bacterial strains), supplemental Table S2 (plasmids), and supplemental Table S3 (primers). All restriction enzymes, T4 DNA ligase, and Antarctic phosphatase were from New England Biolabs. DNA purification kits were from BioBasic (Markham, Ontario, Canada). Genomic DNA for *F. nucleatum* ATCC 25586 was purchased from ATCC (Manassas, VA) and used to create all recombinant FplA constructs for expression described herein. pET16b was used as the base expression vector for *E. coli* expression of FplA constructs. PCR products were then spin column–purified and digested overnight at 37 °C with restriction enzymes described in supplemental Table S3. Digested PCR products were spin column–purified and ligated by T4 DNA ligase into pET16b vector that had been restriction enzyme– and Antarctic phosphatase–treated according to the manufacturer's recommended protocol. Ligations were transformed into Mix & Go! (Zymo Research) competent *E. coli* and plated on LB containing 100 μg/ml carbenicillin (ampicillin), followed by verification of positive clones by restriction digest analysis using purified plasmid. Positive clones were then transformed into LOBSTR RIL (62) *E. coli* cells for protein expression.

Specifically, pDJSVT84 (FplA(20–350)), pDJSVT43 (FplA(20-431)), pDJSVT85 (FplA(60–350)), and pDJSVT82 (FplA(60–431)) all produce proteins with a C-terminal His_6_ tag and are expressed in the cytoplasm because these constructs lack the N-terminal signal sequence used to export FplA through the Sec apparatus in *F. nucleatum*. pDJSVT60 (FplA(20–431) S98A) and pDJSVT61 (FplA(20–431) D243A) were created by using pDJSVT43 as a template for QuikChange mutagenesis PCR. Verification of mutants and all clones was performed by Sanger sequencing (Genewiz). To facilitate the export of FplA to the surface of *E. coli*, a new inducible expression vector was created using pET16b as the backbone by incorporating the signal sequence from the *E. coli* protein OmpA (residues 1–27). In addition, this expression vector (pDJSVT86) contains an N-terminal His_6_ tag that remains on the expressed protein after residues 1–21 from OmpA are cleaved in the periplasm. This effectively creates an inducible vector for the expression of periplasmic and outer membrane proteins in *E. coli* that was customized with GC-rich restriction sites (NotI, KpnI, and XhoI) to facilitate enhanced cloning of AT-rich (74%) genomes, such as *F. nucleatum*. Using the pDJSVT86 expression vector, pDJSVT88 (OmpA(1–27), His_6_, FplA(20–760)) was created and shows efficient export of enzymatically active, full-length FplA to the surface of *E. coli* (Fig. 6).

### FplA protein expression and purification

Briefly, all FplA constructs in LOBSTR RIL (62) *E. coli* cells were grown in Studier autoinduction medium (63) (ZYP-5052, 0.05% glucose, 0.5% lactose, 0.5% glycerol) at 37 °C, 250 rpm shaking, and harvested at 20 h postinoculation by pelleting at 5000 × *g* for 15 min at 4 °C. Pellets were weighed and resuspended in lysis buffer (20 mm Tris, pH 7.5, 20 mm imidazole, 400 mm NaCl, 0.1% BOG, 1 mm PMSF) at 10 ml/g of cell pellet. Bacteria were lysed by using five passes on an EmulsiFlex-C3 homogenizer (Avestin, Mannheim, Germany), followed by removal of insoluble material and unlysed cells by pelleting at 15,000 × *g* for 15 min at 4 °C. The resulting supernatant containing His_6_-tagged FplA constructs was gently stirred with 5 ml of NiCl_2_ charged chelating Sepharose beads (GE Healthcare) for 30 min at 4 °C, followed by washing with 200 ml of wash buffer (20 mm Tris, pH 7.5, 50 mm imidazole, 400 mm NaCl, 0.1% BOG). After washing, FplA was eluted in 10 ml of elution buffer (20 mm Tris, pH 7.5, 250 mm imidazole, 50 mm NaCl, 0.1% BOG). This protein was directly applied to a HiTrap Q FP anion exchange column (FplA construct theoretical isoelectric points: 5.91–6.34) and purified on an ÄKTA Pure system (GE Healthcare) using a linear gradient between Buffer A (20 mm Tris, pH 8, 50 mm NaCl, 0.025% BOG) and Buffer B (20 mm Tris, pH 8, 1 m NaCl, 0.025% BOG). Fractions containing FplA as determined by SDS-PAGE analysis were pooled and further purified on a HiPrep 16/60 Sephacryl S-200 HR size exclusion column (GE Healthcare) in 20 mm Tris, pH 7.5, 150 mm NaCl, 10% glycerol. Protein concentrations were determined using a Qubit fluorimeter and BCA assays according to the manufacturer's recommended protocol. Protein purity was determined using ClearPage 4–20% gradient gels (CBS Scientific) and determined to be >95% pure for all constructs.

### Antibody production and Western blotting to detect FplA

Purified FplA(20–431) was used to create a polyclonal antibody in rabbits (New England Peptide). To purify the antibody, FplA(20–431) was coupled to CNBr-activated Sepharose (Bioworld), and anti-FplA(20–431) antiserum adjusted to pH 8.0 with 20 mm Tris-HCl was passed through the column to bind FplA(20–431) antibodies, followed by extensive washing in PBS and elution in 2.7 ml of 100 mm glycine, pH 2.8. To the eluted antibodies, 0.3 ml of 1 m Tris-HCl, pH 8.5 was added, for a final storage buffer of 10 mm glycine, 100 mm Tris-HCl, pH 8.5.

For Western blot detection of FplA, proteins were separated by SDS-PAGE and subsequently transferred to PVDF membranes, blocked in 20 ml of TBST (20 mm Tris, 150 mm NaCl, 0.1% Tween 20) with 3% BSA for 15 h at 4 °C. After blocking, the membranes were incubated with rabbit anti-FplA antibody (1:10,000 for pure proteins, 1:2500–1:1000 whole cells or lysates) in TBST with 3% BSA for 1 h (70 rpm shaking, 26 °C). After incubating with the primary antibody, the membrane was washed with TBST, followed by incubation with goat anti-rabbit HRP secondary antibody (Cell Signaling) at 1:10,000 dilution in TBST with 3% BSA for 30 min (70-rpm shaking, 26 °C). After the secondary antibody incubation, the membrane was washed in TBST, followed by incubation with ECL-Plus blotting reagents (Pierce) and visualization using Lucent Blue X-ray film (Advansta) developed on an SRX-101A medical film processor (Konica, Tokyo, Japan).

### Development of an F. nucleatum 23726 ΔfplA strain

Single-crossover homologous recombination gene knock-outs of *F. nucleatum* 23726 have been reported previously, although as with all *Fusobacterium* mutagenesis strategies, efficiencies are quite low. Based on a previous method (45), we created an integration plasmid that will not replicate in *F. nucleatum*, therefore only producing antibiotic resistant colonies for strains that incorporate the plasmid directly into the chromosome in the gene of interest during transformation and outgrowth. A central 1000-bp region in the FN1704 (*fplA*) gene in *F. nucleatum* 23726 was amplified from genomic DNA by PCR, digested with EcoRI and SpeI, and ligated into pJIR750 that was digested with the same enzymes and subsequently treated with Antarctic phosphatase. The ligation was transformed into Mix & Go! competent *E. coli* and plated on LB plus 10 μg/ml chloramphenicol, followed by selection of colonies, purification of plasmid DNA, and verification of positive clones by restriction digest analysis. A single positive clone was selected for all future studies, and DNA was initially purified by spin column (BioBasic), followed by additional purification of the DNA using glycogen and methanol precipitation, followed by resuspension in sterile deionized H_2_O.

*F. nucleatum* 23726 was made competent by growing a 5-ml culture to midlog phase (*A*_600_ = 0.4) followed by spinning down cells at 14,000 × *g* for 3 min, removal of medium, and five successive 1-ml washes with ice-cold 10% glycerol in deionized H_2_O. Cells were then resuspended in a final volume of 100 μl of ice-cold 10% glycerol (final *A*_600_ ∼20). Bacteria were transferred to cold 1-mm electroporation cuvettes (Genesee), and 0.5–2.0 μg (concentration >500 ng/μl) of pDJSVT100 plasmid was added immediately before electroporating at 2.0 kV (20 kV/cm), 50 microfarads, 129 ohms, using a BTX Electro Cell Manipulator 600 (Harvard Apparatus). To the cuvette, 1 ml of recovery medium (CBHK, 1 mm MgCl_2_) was added and immediately transferred by syringe into a sterile, anaerobic tube via septum for incubation at 37 °C for 20 h with no shaking. After outgrowth, cells were spun down at 14,000 × *g* for 3 min, medium was removed, and cells were resuspended in 0.1 ml of recovery medium, followed by plating on CBHK plates with 5 μg/ml thiamphenicol and incubation in an anaerobic 37 °C incubator for 2 days for colony growth. ∼5 colonies/μg of DNA were achieved, and the *fplA* gene knock-out was verified by PCR specific to the chromosome and *catP* gene that was incorporated into the genome by the pDJSVT100 KO plasmid (for primers, see supplemental Table S3). In addition, Western blots were used to confirm a loss of FplA protein expression (Figs. 7 and 8).

### Enzymatic assay design, data collection, and FplA kinetics

Initial tests for FplA enzymatic activity were run using the EnzChek Phospholipase A_1_ and EnzChek Phospholipase A_2_ assay kits (Thermo Fisher Scientific) at 1 and 10 μm FplA(20–431), using the manufacturer's protocol (Fig. 4*A*). These assays showed that FplA has PLA_1_ but not PLA_2_ activity, which is consistent with data reported for the homologous enzyme PlpD. We then went on to further characterize its activity by developing a continuous kinetic assay using the PLA_1_-specific substrate PED-A1 (Thermo Fisher Scientific) and determined the full kinetic parameters of FplA with this substrate as reported in Fig. 4 and supplemental Fig. S1. In detail, FplA was used at 1 nm in the reaction and substrate (10 mm stock in 100% DMSO) dilutions (0–10 μm), and reactions were carried out in reaction buffer (50 mm Tris, pH 8.5, 50 mm NaCl, 0.025% BOG). All samples, including controls, contained equal concentrations of DMSO. Reactions were run at 26 °C for 30 min with 3 s of shaking between continuous fluorescent monitoring (excitation = 488 nm, emission = 530 nm) every 2 min on a SpectraMax M5^e^ plate reader (Molecular Devices). Relative fluorescence units measured upon cleavage of substrate ester bonds and release of the acyl chain were converted to the concentration of product (BODIPY® FL C5) created by establishing a standard curve using pure BODIPY® FL C5 (Thermo Fisher Scientific). In all enzymatic reactions, controls containing no protein were run, and the values were subtracted from the reactions containing protein during analysis.

We then developed a continuous fluorescent assay to characterize the phospholipase activity of FplA using the general lipase substrates 4-MuB and 4-MuH (Santa Cruz Biotechnology, Inc.). In detail, FplA was used at 1 nm in the reaction, and substrate (50 mm stock in 100% DMSO) dilutions (0–200 μm) and reactions were carried out in reaction buffer (50 mm Tris, pH 8.5, 50 mm NaCl, 0.025% BOG). All samples, including controls, contained equal concentrations of DMSO (0.4%). Reactions were run at 26 °C for 30 min with 3 s of shaking between continuous fluorescent monitoring (excitation = 360 nm, emission = 449 nm) every 2 min on a SpectraMax M5^e^ plate reader. Relative fluorescence units measured upon cleavage of substrate ester bonds and release of the acyl chain were converted to the concentration of product (4-methylumbeliferone; 4-Mu) created by establishing a standard curve using pure 4-Mu (Sigma-Aldrich).

The steady-state kinetic parameters for each substrate were determined using GraphPad Prism version 6 (GraphPad Software, La Jolla, CA) by fitting the initial rate data (*n* = 2) to the Michaelis–Menten equation,

(Eq. 1) $$v=V_{\max}\left[\text{S}\right]/\left(K_{m}+\left[\text{S}\right]\right)$$

to obtain the values reported in Fig. 2 and supplemental Fig. S3.

### Characterization of FplA inhibitors

We set out to characterize inhibitors that we could use as effective tools to test the role of FplA both *in vitro* and potentially *in vivo* by IC_50_ assays using a variety of inhibitor classes. Inhibitors shown in Fig. 5 and supplemental Fig. S2 were chosen based on their previous classification as inhibitors of a diverse set of phospholipase enzymes: MAFP and PLA_2_ (64); ATFMK, cPLA_2_, and iPLA_2_ (65); IDEFP and fatty acid amide hydrolase (66); palmityl trifluoromethyl ketone, cPLA_2_, and iPLA_2_ (67); ML-211, LYPLA1, and LYPLA2 (68); IDFP, fatty acid amide hydrolase, and monoacylglycerol lipase (69); LY311727 and sPLA_2_ (70); and manoalide, sPLA2, and PLC (71, 72). All inhibitors were purchased from Cayman Chemical.

For potent inhibitors, 0–25 μm concentrations were used in assays, and for compounds found to not inhibit efficiently, the concentration range was 0–100 μm. Inhibitors were diluted into reaction buffer (50 mm Tris, pH 8.5, 50 mm NaCl, 0.025% BOG) containing 10 μm 4-MuH. To initiate the reaction, 1 nm final FplA(20–431) was added, and reactions were run at 26 °C for 30 min with 3 s of shaking between continuous fluorescent monitoring (excitation = 360 nm, emission = 449 nm) every 2 min on a SpectraMax M5^e^ plate reader. Raw data (*n* = 2) for each reaction were analyzed in GraphPad Prism using a log(inhibitor) *versus* response using variable slope and a least squares (ordinary) fit model.

### Use of fluorescent chemical probes to label and detect FplA

Purified recombinant FplA constructs or WT FplA from *F. nucleatum* strains were visualized using an ActivX TAMRA-FP probe (Thermo Fisher Scientific). This probe only binds to proteins with activated serine residues. For purified recombinant proteins, 5 μg of purified protein was incubated with either 100 μm MAFP or PBS for 1 h. Following preincubation with MAFP or PBS, 1 μm ActivX TAMRA-FP probe was added to the protein and incubated for 20 min at 26 °C followed by the addition of SDS-PAGE running buffer to stop the reaction. 500 ng of protein was run on an SDS-polyacrylamide gel at 210 V for 60 min, followed by transfer of proteins to PVDF membranes in transfer buffer (25 mm Tris, 190 mm glycine, 20% methanol, pH 8.3) at 80 V for 60 min. Fluorescent proteins were visualized using a G:Box XX6 system (SynGene) using the TAMRA fluorescence filter.

For the detection of FplA in *F. nucleatum* whole-cell mixtures, 5 ml of *F. nucleatum* 23726 or *F. nucleatum* 23726 Δ*fplA* cells at *A*_600_ = 0.2 were pelleted, washed, and resuspended in 100 μl of PBS. ActivX TAMRA-FP was added at a final concentration of 2 μm and incubated at 26 °C for 20 min, followed by the addition of SDS buffer. 10 μl of this reaction (lysate from ∼4.2 × 10^8^ bacteria) was run per well on an SDS-polyacrylamide gel at 210 V for 60 min. Gels were then imaged on a Typhoon Trio imager (GE Healthcare) using the TAMRA filter setting.

### Detection of FplA on the surface of E. coli by microscopy, enzymatic activity, and proteinase K treatment

Using the expression vector pDJSVT86 that is described under “Cloning of FplA constructs for expression in E. coli,” we cloned FplA(20–760) into the vector at the 3′ end of the OmpA(1–27)-His_6_ signal sequence (pDJSVT88). This construct was expressed in LOBSTR RIL (62) *E. coli* in Studier autoinduction medium at 37 °C for 20 h with 250-rpm shaking. The empty vector pDJSVT86 was used as a negative control for FplA expression for both microscopy and enzymatic assays.

For microscopy, stationary phase bacteria from overnight expressions were washed in PBS, pH 7.5, 0.2% gelatin and spun down at 5000 × *g* for 5 min, followed by resuspension of the bacteria at an *A*_600_ = 0.2. To the bacteria, a final 3.2% paraformaldehyde was added for 15 min at 26 °C for fixation, followed by washing in PBS, pH 7.5, 0.2% gelatin. 500 μl of fixed bacteria were then added on top of a polylysine-coated coverslip in a 6-well plate, and 2 ml of PBS was added for a final volume of 2.5 ml. Bacteria were then spun down onto the coverslips at 2000 × *g* for 10 min. Washed coverslips were submerged in 300 μl of PBS, pH 7.5, 0.2% gelatin containing a 1:100 dilution of the anti-FplA antibody and incubated for 20 h at 26 °C with light shaking. Coverslips were washed again in PBS, pH 7.5, 0.2% gelatin and then incubated in the same buffer containing an anti-rabbit Alexa Fluor 488–conjugated secondary antibody for 30 min at 26 °C. Washed coverslips were mounted with Cytoseal 60 (Thermo Fisher Scientific) and visualized by brightfield and fluorescence microscopy using the GFP channel on an EVOS FL microscope (Life Technologies, Inc.).

For the enzymatic activity assay, stationary phase bacteria from overnight expressions were washed in PBS, pH 7.5, and spun down at 5000 × *g* for 5 min, followed by resuspension of the bacteria at an *A*_600_ = 0.2 in PBS, pH 7.5. Bacterial samples were incubated with 10 μm MAFP or PBS, pH 7.5, at room temperature for 60 min at 26 °C, followed by washing in PBS, pH 7.5, and resuspension to the original *A*_600_ = 0.2 (2 × 10^8^ CFU/ml in enzymatic assay buffer (50 mm Tris, pH 8.5, 50 mm NaCl, 0.025% BOG). 2 × 10^6^ bacteria were then added to reaction wells containing 10 μm 4-MuH fluorescent lipase substrate (excitation = 360 nm, emission 449 nm), followed by incubation at 37 °C for 30 min and detection of lipid cleavage and product formation with a Spectramax M5^e^ as seen in Fig. 6*B*. Activity was plotted as fluorescence units, and statistical analysis was performed using a multiple-comparison analysis by one-way analysis of variance in GraphPad Prism.

To further validate the translocation of the PLA_1_ domain of FplA to the surface of *E. c*oli, the nonspecific and membrane-impenetrable enzyme PK was used to cleave FplA in a dose-dependent manner. FplA expression was induced with 500 μm isopropyl 1-thio-β-d-galactopyranoside for 4 h with shaking at 37 °C. Bacteria were washed in PBS and adjusted to an *A*_600_ = 0.2 in PBS with 1 mm CaCl_2_ to activate PK. 100 μl of cells were added to tubes, followed by the addition of 0, 100, 250, or 1000 nm PK and incubation at 26 °C for 15 min. Reactions were then quenched with protease inhibitors (Roche Applied Science), and samples were separated by SDS-PAGE and transferred to PVDF for Western blot analysis with an anti-FplA antibody. As a control, *E. coli* with the empty vector pDJSVT86 was analyzed for FplA expression and cleavage. In addition, GAPDH was used a load control and also as a control to show that PK was not digesting intracellular proteins.

### Lipid-binding assays

Binding of FplA to various lipids was performed with commercially available lipids spotted on membranes or by our laboratory spotting fresh lipids on blots.

For the first analysis, membrane lipid strips were purchased from Eschelon, Inc. The strips were blocked in 10 ml of TBST with 3% BSA for 2 h at 26 °C with 70-rpm shaking. After blocking, lipid strips were incubated with TBST with 3% BSA containing 50 μg/ml of the indicated FplA construct at 4 °C for 15 h. After incubation with FplA, lipid strips were washed with TBST and incubated with a 1:1000 dilution of rabbit anti-FplA antibody in 10 ml of TBST with 3% BSA for 60 min at 26 °C with 70-rpm shaking. Lipid strips were washed with TBST and incubated with a 1:2000 dilution of goat anti-rabbit IgG-HRP–linked antibody (Cell Signaling) in 10 ml of TBST with 3% BSA for 30 min at 26 °C with 70-rpm shaking. After secondary antibody incubation, the lipid strips were thoroughly washed in TBST, and ECL-Plus blotting reagents were added for visualization (73). The membranes were visualized using a G:Box XX6 system (SynGene) (supplemental Fig. S5).

For a more detailed analysis of FplA binding to phosphoinositides, we purchased various phosphoinositides from Avanti Polar Lipids and then spotted them onto PVDF at concentrations from 0 to 200 pmol. We tested FplA binding to PI, PI(3)P, PI(4)P, PI(5)P, PI(3,4)P_2_, PI(3,5)P_2_, PI(4,5)P_2_, PI(3,4,5)P_3_, and cardiolipin. All steps for analysis were the same as described above, except the membranes were visualized using Lucent Blue X-ray film developed on an SRX-101A medical film processor (Fig. 9).

## Author contributions

M. A. C. and C. C. Y. designed, performed, and analyzed experiments and wrote the manuscript. H. B. S. purified protein and performed kinetic experiments. A. J. D. grew fusobacterium and created the fplA knockout strain. K. C. prepared and sequenced all strains of Fusobacterium used in the study. A. C. V. purified protein and performed kinetic experiments. E. A.-V. analyzed and wrote the manuscript. D. J. S. designed, performed, and analyzed experiments and wrote the manuscript. All authors analyzed the results and approved the final version of the manuscript.

## Supplementary Material

Supplemental Data

## Footnotes

3 M. A. Casasanta, C. C. Yoo, H. B. Smith, A. J. Duncan, K. Cochrane, A. C. Varano, E. Allen-Vercoe, and D. J. Slade, unpublished data.

## Footnotes

2 The abbreviations used are:

PDB: Protein Data Bank
PLA_1_: phospholipase A_1_
4-Mu: 4-methylumbeliferone
4-MuB: 4-methylumbelliferyl butyrate
4-MuH: 4-methylumbelliferyl heptanoate
MAFP: methylarachidonyl fluorophosphonate
ATFMK: arachidonyl trifluoromethyl ketone
IDEFP: isopropyl dodec-11-enylfluorophosphonate
IDFP: isopropyl dodecylfluorophosphonate
BOG: *n*-octyl-β-d-glucopyranoside
PI: phosphoinositide
PI(3)P: phosphoinositide 3-phosphate
PI(4)P: phosphoinositide 4-phosphate
PI(5)P: phosphoinositide 5-phosphate
PI(3,4)P_2_: phosphoinositide 3,4-bisphosphate
PI(3,5)P_2_: phosphoinositide 3,5-bisphosphate
PI(4,5)P_2_: phosphoinositide 4,5-bisphosphate
PI(3,4,5)P_3_: phosphoinositide 3,4,5-trisphosphate.

## References

[1] Signat B., Roques C., Poulet P., and Duffaut D. (2011) *Fusobacterium nucleatum* in periodontal health and disease. Curr. Issues Mol. Biol. 13, 25–36 21220789

[2] Han Y. W., Redline R. W., Li M., Yin L., Hill G. B., and McCormick T. S. (2004) *Fusobacterium nucleatum* induces premature and term stillbirths in pregnant mice: implication of oral bacteria in preterm birth. Infect. Immun. 72, 2272–2279 15039352 10.1128/IAI.72.4.2272-2279.2004 PMC375172

[3] Abed J., Emgård J. E., Zamir G., Faroja M., Almogy G., Grenov A., Sol A., Naor R., Pikarsky E., Atlan K. A., Mellul A., Chaushu S., Manson A. L., Earl A. M., Ou N., et al (2016) Fap2 mediates *Fusobacterium nucleatum* colorectal adenocarcinoma enrichment by binding to tumor-expressed Gal-GalNAc. Cell Host Microbe 20, 215–225 27512904 10.1016/j.chom.2016.07.006 PMC5465824

[4] Dahya V., Patel J., Wheeler M., and Ketsela G. (2015) *Fusobacterium nucleatum* endocarditis presenting as liver and brain abscesses in an immunocompetent patient. Am. J. Med. Sci. 349, 284–285 25588024 10.1097/MAJ.0000000000000388

[5] Rashidi A., Tahhan S. G., Cohee M. W., and Goodman B. M. (2012) *Fusobacterium nucleatum* infection mimicking metastatic cancer. Indian J. Gastroenterol. 31, 198–200 22875742 10.1007/s12664-012-0233-x

[6] Gedik A. H., Cakir E., Soysal O., and Umutoğlu T. (2014) Endobronchial lesion due to pulmonary *Fusobacterium nucleatum* infection in a child. Pediatr. Pulmonol. 49, E63–E65 23868895 10.1002/ppul.22834

[7] Shammas N. W., Murphy G. W., Eichelberger J., Klee D., Schwartz R., and Bachman W. (1993) Infective endocarditis due to *Fusobacterium nucleatum*: case report and review of the literature. Clin. Cardiol. 16, 72–75 8416766 10.1002/clc.4960160116

[8] Swidsinski A., Dörffel Y., Loening-Baucke V., Theissig F., Rückert J. C., Ismail M., Rau W. A., Gaschler D., Weizenegger M., Kühn S., Schilling J., and Dörffel W. V. (2011) Acute appendicitis is characterised by local invasion with *Fusobacterium nucleatum*/necrophorum. Gut 60, 34–40 19926616 10.1136/gut.2009.191320

[9] Han Y.W., (2015) *Fusobacterium nucleatum*: a commensal-turned pathogen. Curr. Opin. Microbiol. 23, 141–147 25576662 10.1016/j.mib.2014.11.013 PMC4323942

[10] Gauthier S., Tétu A., Himaya E., Morand M., Chandad F., Rallu F., and Bujold E. (2011) The origin of *Fusobacterium nucleatum* involved in intra-amniotic infection and preterm birth. J. Matern. Fetal Neonatal Med. 24, 1329–1332 21314291 10.3109/14767058.2010.550977

[11] Castellarin M., Warren R. L., Freeman J. D., Dreolini L., Krzywinski M., Strauss J., Barnes R., Watson P., Allen-Vercoe E., Moore R. A., and Holt R. A. (2012) *Fusobacterium nucleatum* infection is prevalent in human colorectal carcinoma. Genome Res. 22, 299–306 22009989 10.1101/gr.126516.111 PMC3266037

[12] Warren R. L., Freeman D. J., Pleasance S., Watson P., Moore R. A., Cochrane K., Allen-Vercoe E., and Holt R. A. (2013) Co-occurrence of anaerobic bacteria in colorectal carcinomas. Microbiome 1, 16 24450771 10.1186/2049-2618-1-16 PMC3971631

[13] Kostic A. D., Gevers D., Pedamallu C. S., Michaud M., Duke F., Earl A. M., Ojesina A. I., Jung J., Bass A. J., Tabernero J., Baselga J., Liu C., Shivdasani R. A., Ogino S., Birren B. W., et al (2012) Genomic analysis identifies association of *Fusobacterium* with colorectal carcinoma. Genome Res. 22, 292–298 22009990 10.1101/gr.126573.111 PMC3266036

[14] Kostic A. D., Chun E., Robertson L., Glickman J. N., Gallini C. A., Michaud M., Clancy T. E., Chung D. C., Lochhead P., Hold G. L., El-Omar E. M., Brenner D., Fuchs C. S., Meyerson M., and Garrett W. S. (2013) *Fusobacterium nucleatum* potentiates intestinal tumorigenesis and modulates the tumor-immune microenvironment. Cell Host Microbe 14, 207–215 23954159 10.1016/j.chom.2013.07.007 PMC3772512

[15] Flanagan L., Schmid J., Ebert M., Soucek P., Kunicka T., Liska V., Bruha J., Neary P., Dezeeuw N., Tommasino M., Jenab M., Prehn J. H., and Hughes D. J. (2014) *Fusobacterium nucleatum* associates with stages of colorectal neoplasia development, colorectal cancer and disease outcome. Eur. J. Clin. Microbiol. Infect. Dis. 33, 1381–1390 24599709 10.1007/s10096-014-2081-3

[16] Strauss J., Kaplan G. G., Beck P. L., Rioux K., Panaccione R., Devinney R., Lynch T., and Allen-Vercoe E. (2011) Invasive potential of gut mucosa-derived Fusobacterium nucleatum positively correlates with IBD status of the host. Inflamm. Bowel Dis. 17, 1971–1978 21830275 10.1002/ibd.21606

[17] Manson McGuire A., Cochrane K., Griggs A. D., Haas B. J., Abeel T., Zeng Q., Nice J. B., MacDonald H., Birren B. W., Berger B. W., Allen-Vercoe E., and Earl A. M. (2014) Evolution of invasion in a diverse set of *Fusobacterium* species. MBio 5, e01864 25370491 10.1128/mBio.01864-14 PMC4222103

[18] Rubinstein M. R., Wang X., Liu W., Hao Y., Cai G., and Han Y. W. (2013) *Fusobacterium nucleatum* promotes colorectal carcinogenesis by modulating E-cadherin/β-catenin signaling via its FadA adhesin. Cell Host Microbe 14, 195–206 23954158 10.1016/j.chom.2013.07.012 PMC3770529

[19] Xu M., Yamada M., Li M., Liu H., Chen S. G., and Han Y. W. (2007) FadA from *Fusobacterium nucleatum* utilizes both secreted and nonsecreted forms for functional oligomerization for attachment and invasion of host cells. J. Biol. Chem. 282, 25000–25009 17588948 10.1074/jbc.M611567200

[20] Kaplan C. W., Ma X., Paranjpe A., Jewett A., Lux R., Kinder-Haake S., and Shi W. (2010) *Fusobacterium nucleatum* outer membrane proteins Fap2 and RadD induce cell death in human lymphocytes. Infect. Immun. 78, 4773–4778 20823215 10.1128/IAI.00567-10 PMC2976331

[21] Gur C., Ibrahim Y., Isaacson B., Yamin R., Abed J., Gamliel M., Enk J., Bar-On Y., Stanietsky-Kaynan N., Coppenhagen-Glazer S., Shussman N., Almogy G., Cuapio A., Hofer E., Mevorach D., et al (2015) Binding of the Fap2 protein of *Fusobacterium nucleatum* to human inhibitory receptor TIGIT protects tumors from immune cell attack. Immunity 42, 344–355 25680274 10.1016/j.immuni.2015.01.010 PMC4361732

[22] Coppenhagen-Glazer S., Sol A., Abed J., Naor R., Zhang X., Han Y. W., and Bachrach G. (2015) Fap2 of *Fusobacterium nucleatum* is a galactose-inhibitable adhesin involved in coaggregation, cell adhesion, and preterm birth. Infect. Immun. 83, 1104–1113 25561710 10.1128/IAI.02838-14 PMC4333458

[23] Kaplan C. W., Lux R., Haake S. K., and Shi W. (2009) The *Fusobacterium nucleatum* outer membrane protein RadD is an arginine-inhibitable adhesin required for inter-species adherence and the structured architecture of multispecies biofilm. Mol. Microbiol. 71, 35–47 19007407 10.1111/j.1365-2958.2008.06503.x PMC2741168

[24] Gupta S., Ghosh S. K., Scott M. E., Bainbridge B., Jiang B., Lamont R. J., McCormick T. S., and Weinberg A. (2010) *Fusobacterium nucleatum*-associated β-defensin inducer (FAD-I): identification, isolation, and functional evaluation. J. Biol. Chem. 285, 36523–36531 20847052 10.1074/jbc.M110.133140 PMC2978580

[25] Bhattacharyya S., Ghosh S. K., Shokeen B., Eapan B., Lux R., Kiselar J., Nithianantham S., Young A., Pandiyan P., McCormick T. S., and Weinberg A. (2016) FAD-I: a *Fusobacterium nucleatum* cell wall-associated diacylated lipoprotein that mediates human β defensin 2 induction through Toll-like receptor-1/2 (TLR-1/2) and TLR-2/6. Infect. Immun. 84, 1446–1456 26930710 10.1128/IAI.01311-15 PMC4862701

[26] Desvaux M., Khan A., Beatson S. A., Scott-Tucker A., and Henderson I. R. (2005) Protein secretion systems in *Fusobacterium nucleatum*: genomic identification of Type 4 piliation and complete Type V pathways brings new insight into mechanisms of pathogenesis. Biochim. Biophys. Acta 1713, 92–112 15993836 10.1016/j.bbamem.2005.05.002

[27] Henderson I. R., and Nataro J. P. (2001) Virulence functions of autotransporter proteins. Infect. Immun. 69, 1231–1243 11179284 10.1128/IAI.69.3.1231-1243.2001 PMC98013

[28] Wells T. J., Tree J. J., Ulett G. C., and Schembri M. A. (2007) Autotransporter proteins: novel targets at the bacterial cell surface. FEMS Microbiol. Lett. 274, 163–172 17610513 10.1111/j.1574-6968.2007.00833.x

[29] Fan E., Chauhan N., Udatha D. B. R. K. G., Leo J. C., and Linke D. (2016) Type V secretion systems in bacteria. Microbiol. Spectr. 10.1128/microbiolspec.VMBF-0009-2015 26999388

[30] Albenne C., and Ieva R. (2017) Job contenders: roles of the β-barrel assembly machinery and the translocation and assembly module in autotransporter secretion. Mol. Microbiol. 10.1111/mmi.13832 28887826

[31] Rossiter A. E., Leyton D. L., Tveen-Jensen K., Browning D. F., Sevastsyanovich Y., Knowles T. J., Nichols K. B., Cunningham A. F., Overduin M., Schembri M. A., and Henderson I. R. (2011) The essential β-barrel assembly machinery complex components BamD and BamA are required for autotransporter biogenesis. J. Bacteriol. 193, 4250–4253 21665980 10.1128/JB.00192-11 PMC3147704

[32] Salacha R., Kovacić F., Brochier-Armanet C., Wilhelm S., Tommassen J., Filloux A., Voulhoux R., and Bleves S. (2010) The *Pseudomonas aeruginosa* patatin-like protein PlpD is the archetype of a novel Type V secretion system. Environ. Microbiol. 12, 1498–1512 20192961 10.1111/j.1462-2920.2010.02174.x

[33] da Mata Madeira P. V., Zouhir S., Basso P., Neves D., Laubier A., Salacha R., Bleves S., Faudry E., Contreras-Martel C., and Dessen A. (2016) Structural basis of lipid targeting and destruction by the Type V secretion system of *Pseudomonas aeruginosa*. J. Mol. Biol. 428, 1790–1803 27012424 10.1016/j.jmb.2016.03.012

[34] Pizarro-Cerdá J., and Cossart P. (2004) Subversion of phosphoinositide metabolism by intracellular bacterial pathogens. Nat. Cell Biol. 6, 1026–1033 15516995 10.1038/ncb1104-1026

[35] Istivan T. S., and Coloe P. J. (2006) Phospholipase A in Gram-negative bacteria and its role in pathogenesis. Microbiology 152, 1263–1274 16622044 10.1099/mic.0.28609-0

[36] Flores-Díaz M., Monturiol-Gross L., Naylor C., Alape-Girón A., and Flieger A. (2016) Bacterial sphingomyelinases and phospholipases as virulence factors. Microbiol. Mol. Biol. Rev. 80, 597–628 27307578 10.1128/MMBR.00082-15 PMC4981679

[37] Tannaes T., Bukholm I. K., and Bukholm G. (2005) High relative content of lysophospholipids of *Helicobacter pylori* mediates increased risk for ulcer disease. FEMS Immunol. Med. Microbiol. 44, 17–23 15780574 10.1016/j.femsim.2004.10.003

[38] Dorrell N., Martino M. C., Stabler R. A., Ward S. J., Zhang Z. W., McColm A. A., Farthing M. J., and Wren B. W. (1999) Characterization of *Helicobacter pylori* PldA, a phospholipase with a role in colonization of the gastric mucosa. Gastroenterology 117, 1098–1104 10535872 10.1016/s0016-5085(99)70394-x

[39] Birmingham C. L., Canadien V., Gouin E., Troy E. B., Yoshimori T., Cossart P., Higgins D. E., and Brumell J. H. (2007) *Listeria monocytogenes* evades killing by autophagy during colonization of host cells. Autophagy 3, 442–451 17568179 10.4161/auto.4450

[40] Biasini M., Bienert S., Waterhouse A., Arnold K., Studer G., Schmidt T., Kiefer F., Gallo Cassarino T., Bertoni M., Bordoli L., and Schwede T. (2014) SWISS-MODEL: modelling protein tertiary and quaternary structure using evolutionary information. Nucleic Acids Res. 42, W252–W258 24782522 10.1093/nar/gku340 PMC4086089

[41] Lio Y. C., Reynolds L. J., Balsinde J., and Dennis E. A. (1996) Irreversible inhibition of Ca^2+^-independent phospholipase A2 by methyl arachidonyl fluorophosphonate. Biochim. Biophys. Acta 1302, 55–60 8695655 10.1016/0005-2760(96)00002-1

[42] Liu Y., Patricelli M. P., and Cravatt B. F. (1999) Activity-based protein profiling: the serine hydrolases. Proc. Natl. Acad. Sci. U.S.A. 96, 14694–14699 10611275 10.1073/pnas.96.26.14694 PMC24710

[43] Simon G. M., and Cravatt B. F. (2010) Activity-based proteomics of enzyme superfamilies: serine hydrolases as a case study. J. Biol. Chem. 285, 11051–11055 20147750 10.1074/jbc.R109.097600 PMC2856978

[44] Dekker N., Tommassen J., Lustig A., Rosenbusch J. P., and Verheij H. M. (1997) Dimerization regulates the enzymatic activity of *Escherichia coli* outer membrane phospholipase A. J. Biol. Chem. 272, 3179–3184 9013551 10.1074/jbc.272.6.3179

[45] Kinder Haake S., Yoder S., and Gerardo S. H. (2006) Efficient gene transfer and targeted mutagenesis in *Fusobacterium nucleatum*. Plasmid 55, 27–38 16115683 10.1016/j.plasmid.2005.06.002 PMC1592470

[46] Bannam T. L., and Rood J. I. (1993) *Clostridium perfringens-Escherichia coli* shuttle vectors that carry single antibiotic resistance determinants. Plasmid 29, 233–235 8356117 10.1006/plas.1993.1025

[47] Hug L. A., Baker B. J., Anantharaman K., Brown C. T., Probst A. J., Castelle C. J., Butterfield C. N., Hernsdorf A. W., Amano Y., Ise K., Suzuki Y., Dudek N., Relman D. A., Finstad K. M., Amundson R., et al (2016) A new view of the tree of life. Nat. Microbiol. 1, 16048 27572647 10.1038/nmicrobiol.2016.48

[48] Wilson M. M., Anderson D. E., and Bernstein H. D. (2015) Analysis of the outer membrane proteome and secretome of *Bacteroides fragilis* reveals a multiplicity of secretion mechanisms. PLoS One 10, e0117732 25658944 10.1371/journal.pone.0117732 PMC4319957

[49] Kapatral V., Anderson I., Ivanova N., Reznik G., Los T., Lykidis A., Bhattacharyya A., Bartman A., Gardner W., Grechkin G., Zhu L., Vasieva O., Chu L., Kogan Y., Chaga O., et al (2002) Genome sequence and analysis of the oral bacterium *Fusobacterium nucleatum* strain ATCC 25586. J. Bacteriol. 184, 2005–2018 11889109 10.1128/JB.184.7.2005-2018.2002 PMC134920

[50] Howell H. A., Logan L. K., and Hauser A. R. (2013) Type III secretion of ExoU is critical during early *Pseudomonas aeruginosa* pneumonia. MBio 4, e00032–13 23481600 10.1128/mBio.00032-13 PMC3604777

[51] Machado G-B. S., de Assis M. C., Leão R., Saliba A. M., Silva M. C., Suassuna J. H., de Oliveira A. V., and Plotkowski M. C. (2010) ExoU-induced vascular hyperpermeability and platelet activation in the course of experimental *Pseudomonas aeruginosa* pneumosepsis. Shock 33, 315–321 19543153 10.1097/SHK.0b013e3181b2b0f4

[52] Allewelt M., Coleman F. T., Grout M., Priebe G. P., and Pier G. B. (2000) Acquisition of expression of the *Pseudomonas aeruginosa* ExoU cytotoxin leads to increased bacterial virulence in a murine model of acute pneumonia and systemic spread. Infect. Immun. 68, 3998–4004 10858214 10.1128/iai.68.7.3998-4004.2000 PMC101680

[53] Afra K., Laupland K., Leal J., Lloyd T., and Gregson D. (2013) Incidence, risk factors, and outcomes of *Fusobacterium* species bacteremia. BMC Infect. Dis. 13, 264 23734900 10.1186/1471-2334-13-264 PMC3679863

[54] Heckmann J. G., Lang C. J. G., Hartl H., and Tomandl B. (2003) Multiple brain abscesses caused by *Fusobacterium nucleatum* treated conservatively. Can. J. Neurol. Sci. 30, 266–268 12945954 10.1017/s0317167100002717

[55] Ahmed Z., Bansal S. K., and Dhillon S. (2015) Pyogenic liver abscess caused by *Fusobacterium* in a 21-year-old immunocompetent male. World J. Gastroenterol. 21, 3731–3735 25834342 10.3748/wjg.v21.i12.3731 PMC4375599

[56] Brook I. (2008) Infective endocarditis caused by anaerobic bacteria. Arch. Cardiovasc. Dis. 101, 665–676 19056073 10.1016/j.acvd.2008.08.008

[57] Muyzer G., de Waal E. C., and Uitterlinden A. G. (1993) Profiling of complex microbial populations by denaturing gradient gel electrophoresis analysis of polymerase chain reaction-amplified genes coding for 16S rRNA. Appl. Environ. Microbiol. 59, 695–700 7683183 10.1128/aem.59.3.695-700.1993 PMC202176

[58] Hyatt D., Chen G. L., Locascio P. F., Land M. L., Larimer F. W., and Hauser L. J. (2010) Prodigal: prokaryotic gene recognition and translation initiation site identification. BMC Bioinformatics 11, 119 20211023 10.1186/1471-2105-11-119 PMC2848648

[59] Eddy S. R. (1998) Profile hidden Markov models. Bioinformatics 14, 755–763 9918945 10.1093/bioinformatics/14.9.755

[60] Kearse M., Moir R., Wilson A., Stones-Havas S., Cheung M., Sturrock S., Buxton S., Cooper A., Markowitz S., Duran C., Thierer T., Ashton B., Meintjes P., and Drummond A. (2012) Geneious Basic: an integrated and extendable desktop software platform for the organization and analysis of sequence data. Bioinformatics 28, 1647–1649 22543367 10.1093/bioinformatics/bts199 PMC3371832

[61] Arnold K., Bordoli L., Kopp J., and Schwede T. (2006) The SWISS-MODEL workspace: a web-based environment for protein structure homology modelling. Bioinformatics 22, 195–201 16301204 10.1093/bioinformatics/bti770

[62] Andersen K. R., Leksa N. C., and Schwartz T. U. (2013) Optimized *E. coli* expression strain LOBSTR eliminates common contaminants from His-tag purification. Proteins 81, 1857–1861 23852738 10.1002/prot.24364 PMC4086167

[63] Studier F. W. (2005) Protein production by auto-induction in high density shaking cultures. Protein Expr. Purif. 41, 207–234 15915565 10.1016/j.pep.2005.01.016

[64] Huang Z., Liu S., Street I., Laliberte F., and Abdullah K. (1994) Methyl arachidonyl fluorophosphonate, a potent irreversible cPLA2 inhibitor, blocks the mobilization of arachidonic acid in human platelets and neutrophils. Mediators Inflamm. 3, 307–308

[65] Street I. P., Lin H. K., Laliberté F., Ghomashchi F., Wang Z., Perrier H., Tremblay N. M., Huang Z., Weech P. K., and Gelb M. H. (1993) Slow- and tight-binding inhibitors of the 85-kDa human phospholipase A2. Biochemistry 32, 5935–5940 8018213 10.1021/bi00074a003

[66] Segall Y., Quistad G. B., Sparks S. E., Nomura D. K., and Casida J. E. (2003) Toxicological and structural features of organophosphorus and organosulfur cannabinoid CB1 receptor ligands. Toxicol. Sci. 76, 131–137 12944586 10.1093/toxsci/kfg216

[67] Ackermann E. J., Conde-Frieboes K., and Dennis E. A. (1995) Inhibition of Macrophage Ca^2+^-independent phospholipase A by bromoenol lactone and trifluoromethyl ketones. J. Biol. Chem. 270, 445–450 7814408 10.1074/jbc.270.1.445

[68] Adibekian A., Martin B. R., Speers A. E., Brown S. J., and Spicer T. (2013) Optimization and characterization of a triazole urea dual inhibitor for lysophospholipase 1 (LYPLA1) and lysophospholipase 2 (LYPLA2). Probe Reports from the NIH Molecular Libraries Program, National Center for Biotechnology Information, Bethesda, MD 23658947

[69] Nomura D. K., Blankman J. L., Simon G. M., Fujioka K., Issa R. S., Ward A. M., Cravatt B. F., and Casida J. E. (2008) Activation of the endocannabinoid system by organophosphorus nerve agents. Nat. Chem. Biol. 4, 373–378 18438404 10.1038/nchembio.86 PMC2597283

[70] Schevitz R. W., Bach N. J., Carlson D. G., Chirgadze N. Y., Clawson D. K., Dillard R. D., Draheim S. E., Hartley L. W., Jones N. D, and Mihelich E. D. (1995) Structure-based design of the first potent and selective inhibitor of human non-pancreatic secretory phospholipase A2. Nat. Struct. Biol. 2, 458–465 7664108 10.1038/nsb0695-458 PMC7097651

[71] Randazzo A., Debitus C., Minale L., García Pastor P., Alcaraz M. J., Payá M., and Gomez-Paloma L. (1998) Petrosaspongiolides M-R: new potent and selective phospholipase A2 inhibitors from the New Caledonian marine sponge *Petrosaspongia nigra*. J. Nat. Prod. 61, 571–575 9599251 10.1021/np9704922

[72] Bennett C. F., Mong S., Wu H. L., Clark M. A., Wheeler L., and Crooke S. T. (1987) Inhibition of phosphoinositide-specific phospholipase C by manoalide. Mol. Pharmacol. 32, 587–593 3683364

[73] Perez Y., Maffei M., Amata I., Arbesú M., and Pons M. (2013) Lipid binding by disordered proteins. Protocol Exchange 10.1038/protex.2013.094

