# Supplementary material for: A chemical and biological toolbox for Type Vd secretion: Characterization of the phospholipase A1 autotransporter FplA from Fusobacterium nucleatum
Source: J Biol Chem. 2017 Oct 11;292(49):20240–54. doi: 10.1074/jbc.M117.819144 (PMC5724010; doi:10.1074/jbc.M117.819144)
Supplement: Supplemental Data [file supp_292_49_20240__index.html]

A chemical and biological toolbox for Type Vd secretion: Characterization of the phospholipase A1 autotransporter FplA from Fusobacterium nucleatum — A chemical and biological toolbox for Type Vd secretion: Characterization of the phospholipase A1 autotransporter FplA from Fusobacterium nucleatum — A chemical and biological toolbox for Type Vd secretion — Supplemental Data 

# A chemical and biological toolbox for Type Vd secretion: Characterization of the phospholipase A1 autotransporter FplA from *Fusobacterium nucleatum*

## Supplemental Data

- supplemental (.pdf, 2.4 MB) - supplemental
