# Supplementary material for: A chemical and biological toolbox for Type Vd secretion: Characterization of the phospholipase A1 autotransporter FplA from Fusobacterium nucleatum
Source: J Biol Chem. 2017 Oct 11;292(49):20240–54. doi: 10.1074/jbc.M117.819144 (PMC5724010; doi:10.1074/jbc.M117.819144)
Supplement: Supplemental Data [file 10.1074_M117.819144_jbc.M117.819144-1.pdf]

Figure S1

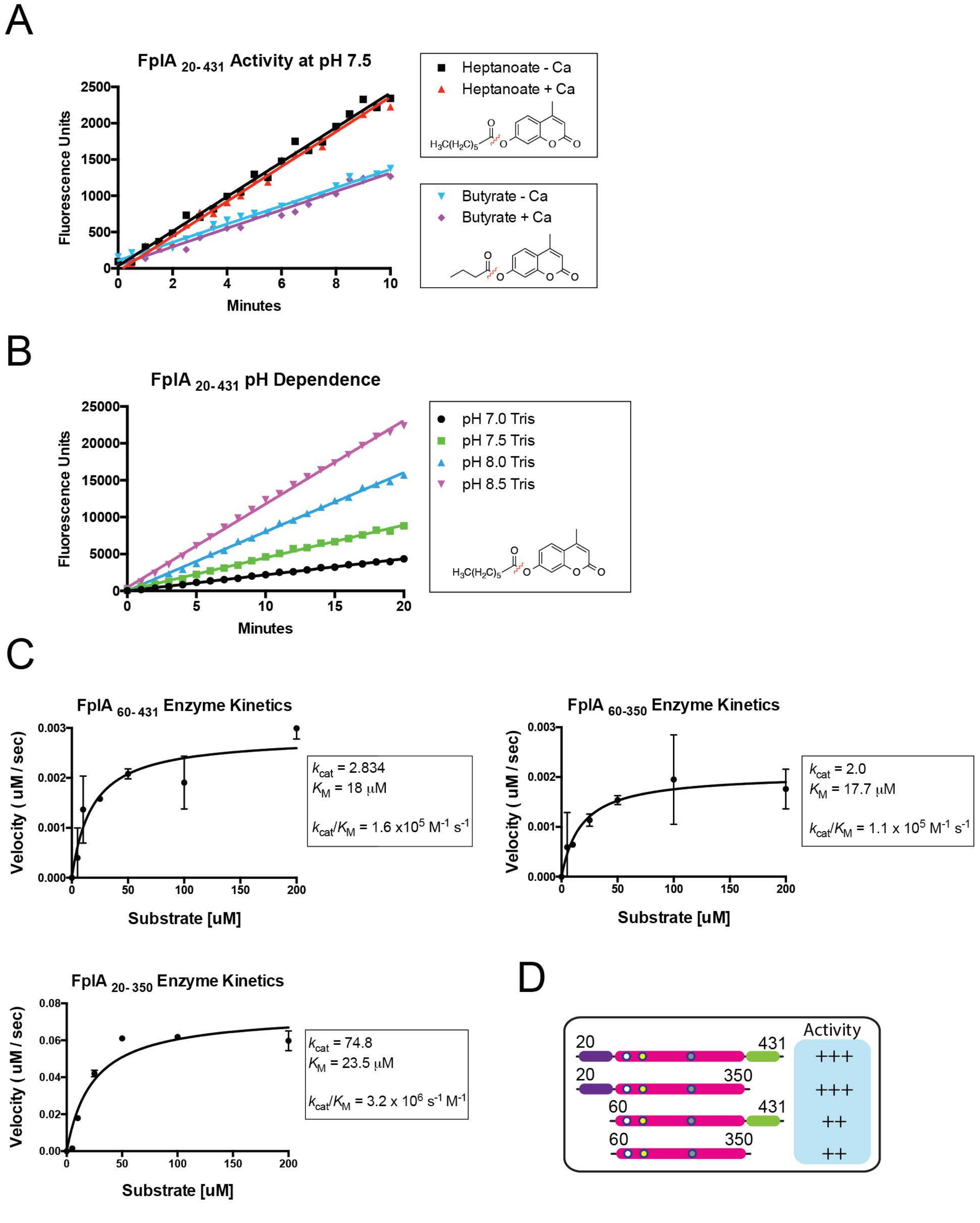

Fig S1 Enzymatic analysis of FplA$_{20\text{-}431}$. (A) FplA$_{20\text{-}431}$ does not need calcium for activity and is more active against the substrate 4-MuH than 4-MuB, indicating that longer acyl chains are critical for substrate binding. (B) pH dependent active of FplA$_{20\text{-}431}$ using 4-MuH as a substate shows maximal activity at pH 8.5. (C) Eznyme kinetics and activity plots of FplA$_{60\text{-}431,}$ FplA$_{60\text{-}350,}$ and FplA$_{20\text{-}350}$ with 4-MuH. (D) Graphic representation of FplA construct activity and the importance of the N-terminal extension (NTE).

Figure S2

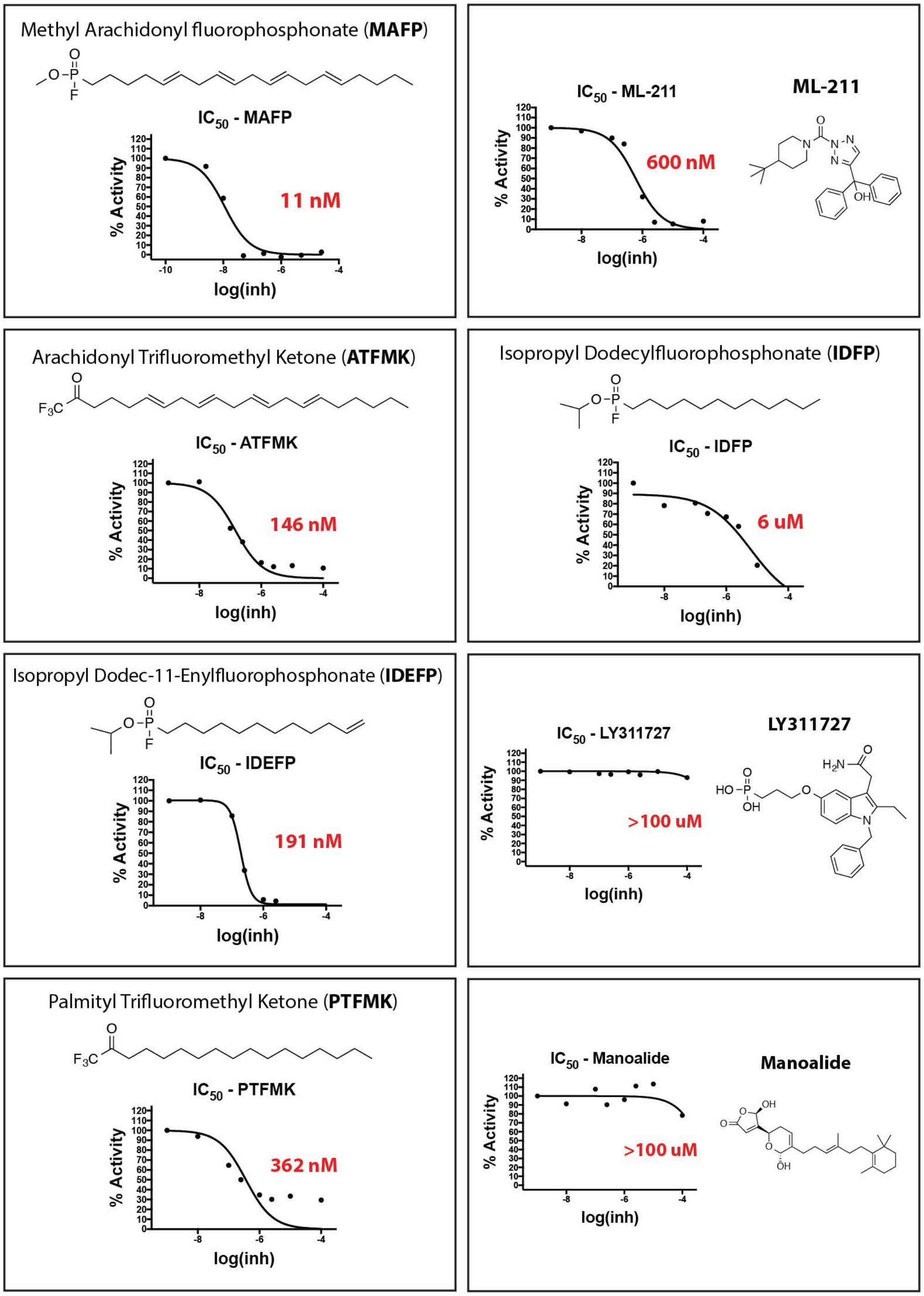

Fig S2 Analysis of multiple inhibitors previously shown to inhibit a diverse set of phospholipases. IC_{50} values equte to the concentration of inhibitor necessary to achieve 50% inhibition of $FplA_{20\text{-}431}$ using 4-MuH as a substrate.

Figure S3

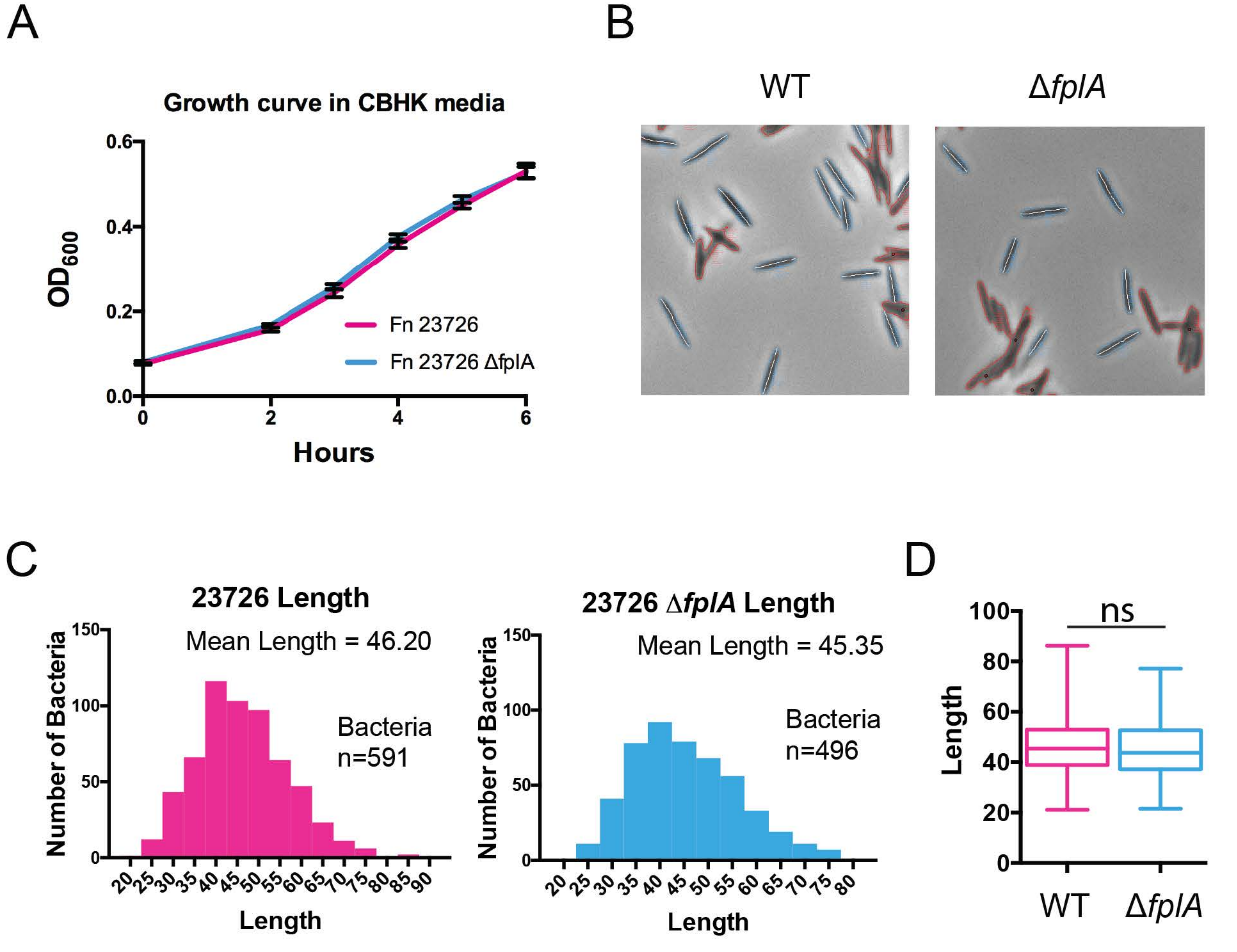

Fig S3 (A) Analysis of a Δ*fplA* mutant on *F. nucleatum* 23726 growth in rich CBHK media reveals no growth defect in the abscence of FplA. (B-D) Loss of FplA does not alter bacterial cell shape or length as determined by analyzing n=~500 bacteria using the MicrobeJ plugin for ImageJ. Statistical analysis was performed using an unpaired t test. ns = not significant (p-value = 0.195).

Figure S4

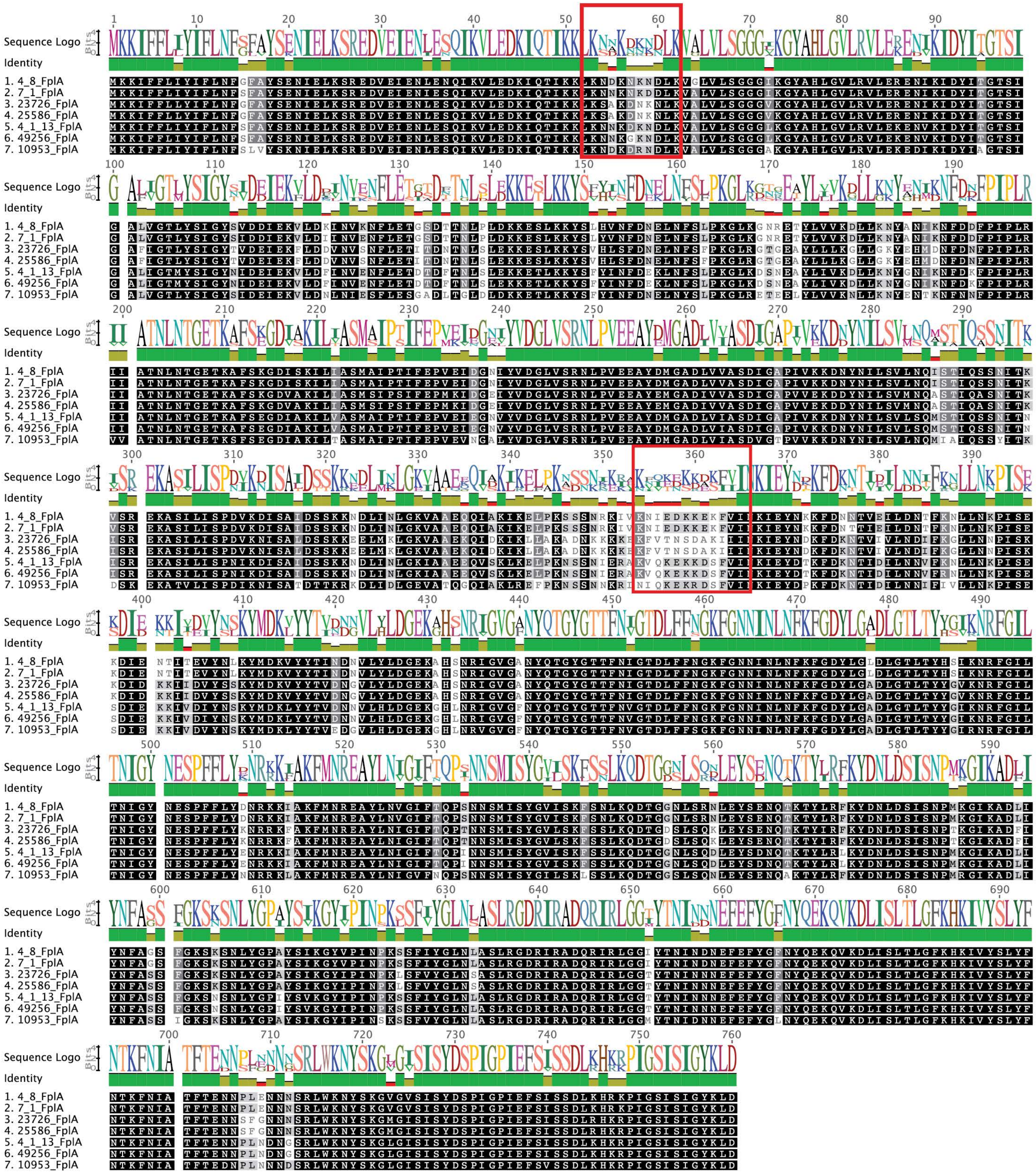

Fig S4 Alignment of full-length FplA from 7 strains of *Fusobacterium*. Potential cleavage sites for release of the PLA_1 domain are outlined in red and were determined based on differences seen in the 23726 and 25586 strains (not cleaved) when compared to the remaining five strains that are cleaved.

Figure S5

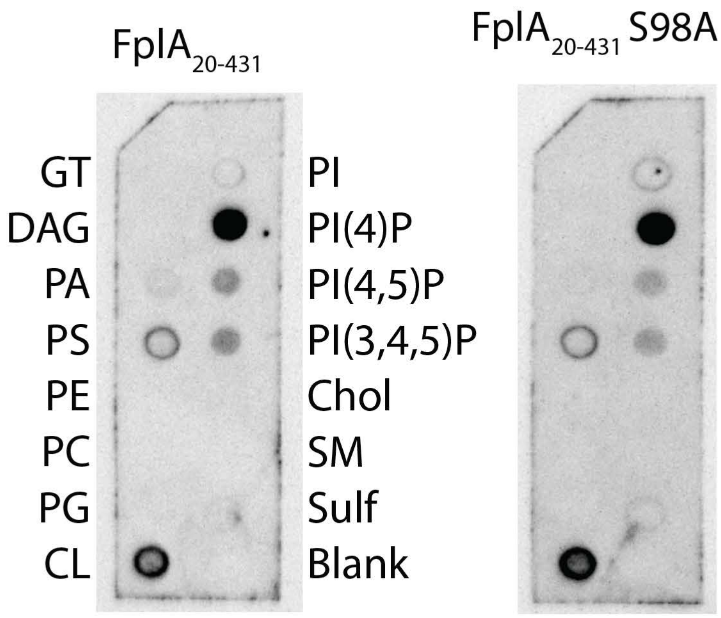

Fig S5 Initial analysis of FplA$_{20\text{-}431}$ and FplA$_{20\text{-}431}$ S98A lipid binding using commercially available lipid strips from Eschelon Inc. Subsequent analysis used freshly blotted lipids (Avanti Polar Lipids) and revealed strong affinity for phosphoinsositides, but not PI(4)p as seen here (see Figure 9).

Supplementary Table 1: Bacterial strains used in this study (Casasanta et al).

Strain	Bacterial Species	Relevant genotype	Source or Reference
TOP10	*E. coli*	*mcrA*, Δ(*mrr-hsdRMS-mcrBC*), Phi80(*del*)*M15*, Δl*acX74*, *deoR*, *recA1*, *araD139*, Δ(*ara-leu*)7697, *galU*, *galK*, *rpsL*(*SmR*), *endA1*, *nupG*	Invitrogen
LOBSTR-BL21(DE3)-RIL	*E. coli*	*fhuA2* [*lon*] *ompT gal* (*λ DE3*) [*dcm*] ∆*hsdS* λ DE3 = λ *sBamHIo* ∆*EcoRI-B int::(lacI::PlacUV5::T7 gene1) i21* ∆*nin5 arnA*(H359S, H361S, H592S, H593S) *slyD*(1-150)	[1]
F. nucleatum nucleatum ATCC 23726	*F. nucleatum*	Wild Type	ATCC, [2–5]
DJSVT01	*F. nucleatum*	*F. nucleatum* ATCC 23726 Cm^r Tm^r ΔFN1704::*catP*	This Study
F. nucleatum nucleatum ATCC 25586	*F. nucleatum*	Wild Type	ATCC, [2–5]
F. nucleatum polymorphum 10953	*F. nucleatum*	Wild Type	ATCC, [2–5]
F. nucleatum vincentii ATCC 49256	*F. nucleatum*	Wild Type	ATCC, [2–5]
F. nucleatum vincentii 4_1_13	*F. nucleatum*	Wild Type	[5]
F. nucleatum animalis 4_8	*F. nucleatum*	Wild Type	[5]
F. nucleatum animalis 7_1	*F. nucleatum*	Wild Type	[5]

Cm^r, Chloramphenicol resistance

Tm^r, Thiamphenicol resistance

1. Andersen KR, Leksa NC, Schwartz TU. Optimized E. coli expression strain LOBSTR eliminates common contaminants from His-tag purification. Proteins. 2013;81: 1857–1861.

2. Knorr M. Über die fusospirilläre Symbiose, die Gattung Fusobacterium (KB Lehmann) und Spirillum sputigenum. Zugleich ein Beiträg zür Bakteriologie der Mundhohle. II. Mitteilung Die Gattung Fusobacterium I Abt Orig Zentralbl Bakteriol Parasitenkd Infektionskr Hyg. 1922;89: 4–22.

3. Knorr M. Ueber die fusospirilläre symbiose, die Gattung Fusobacterium (KB Lehmann) und Spirillum sputigenum. II Mitteilung. Die Gattung Fusobacterium. Zentbl Bakteriol Parasitenkd Infekt Hyg Abt. 1923;1: 4–22.

4. Dzink JL, Sheenan MT, Socransky SS. Proposal of three subspecies of Fusobacterium nucleatum Knorr 1922: Fusobacterium nucleatum subsp. nucleatum subsp. nov., comb. nov.; Fusobacterium nucleatum subsp. polymorphum subsp. nov., nom. rev., comb. nov.; and Fusobacterium nucleatum subsp. vincentii subsp. nov., nom. rev., comb. nov. Int J Syst Evol Microbiol. Microbiology Society; 1990;40: 74–78.

5. Manson McGuire A, Cochrane K, Griggs AD, Haas BJ, Abeel T, Zeng Q, et al. Evolution of invasion in a diverse set of Fusobacterium species. MBio. 2014;5: e01864.

Supplementary Table 2: Plasmids used in this study (Casasanta et al).

Plasmid	Description	Reference
pET16b	*E. coli* inducible expression vector	EMD Millipore
pJIR750	Base *C. perfringens-E. coli* shuttle vector to make *F. nucleatum* shuttle vectors.	[1]
pDJSVT43	*F. nucleatum* 25586 FN1704 (*fplA*) 20-431 C-6xHis cloned into pET16b	This Study
pDJSVT60	*F. nucleatum* 25586 FN1704 (*fplA*) 20-431 S98A C-6xHis cloned into pET16b	This Study
pDJSVT61	*F. nucleatum* 25586 FN1704 (*fplA*) 20-431 D243A C-6xHis cloned into pET16b	This Study
pDJSVT82	*F. nucleatum* 25586 FN1704 (*fplA*) 60-431 C-6xHis cloned into pET16b	This Study
pDJSVT84	*F. nucleatum* 25586 FN1704 (*fplA*) 20-350 C-6xHis cloned into pET16b	This Study
pDJSVT85	*F. nucleatum* 25586 FN1704 (*fplA*) 60-350 C-6xHis cloned into pET16b	This Study
pDJSVT86	*E. coli* inducible expression vector - pET16b with *E.coli* OmpA signal sequence (residues 1-27) followed by a 6xHis tag and multiple cloning site.	This Study
pDJSVT88	*F. nucleatum* 25586 FN1704 (*fplA*) 20-760 cloned into pDJSVT86 for expression of full length FplA on the surface of *E. coli*	This Study
pDJSVT100	Shuttle vector to make strain DJSVT01 (*Table S1*): *F. nucleatum* ATCC 23726 ΔFN1704::*catP* (Cm^r Tm^r)	This Study

Cm^r, Chloramphenicol resistance

Tm^r, Thiamphenicol resistance

1. Bannam TL, Rood JI. Clostridium perfringens-Escherichia coli shuttle vectors that carry single antibiotic resistance determinants. Plasmid. 1993;29: 233–235.

Supplementary Table 3: Primers introduced in this study (Casasanta et al).

Primer	Sequence 5' to 3'	Direction	Restriction Site	Tag	FplA Construct	Strain/Plasmid (*Table S1, S2*)
prDJSVT95	GCACTACCCATGGAAAATATCGAATTAAAATCAAGAG	Forward	NcoI		AA 20-350, 20-431	pDJSVT43, pDJSVT84
prDJSVT96	CTAGTTCTCGAGTTAATGATGATGATGATGATGTGCTTTTTCTCCATCTAAATATAAAAC	Reverse	XhoI	6xHis	AA 20-431, 60-431	pDJSVT43, pDJSVT82
prDJSVT135	CTATATAACAGGTACTGCTATAGGAGCCTTTATTG	Forward			QuikChange: AA 20-431 S98A	pDJSVT60
prDJSVT136	CAATAAAGGCTCCTATAGCAGTACCTGTTATATAG	Reverse			QuikChange: AA 20-431 S98A	pDJSVT60
prDJSVT137	GGAAATATATGTTGCTGGTCTTGTTAGTAG	Forward			QuikChange: AA 20-431 D243A	pDJSVT61
prDJSVT138	CTACTAACAAGACCAGCAACATATATTTCC	Reverse			QuikChange: AA 20-431 D243A	pDJSVT61
prDJSVT153	CTAGTTCTCGAGTTAATCTAATTTATATCCAATTG	Reverse	XhoI		OmpA 1-27 FplA 20-760	pDJSVT88
prDJSVT203	GCACTACGCGGCCGCGGAAAATATCGAATTAAAATCAAGAG	Forward	NotI		OmpA 1-27 FplA 20-760	pDJSVT88
prDJSVT211	GCACTACCCATGGGAAATTTAAAAGTTGCTCTAGTTTTAAG	Forward	NcoI		AA 60-350, 60-431	pDJSVT82
prDJSVT214	CTAGTCTCGAGTTAGTGGTGGTGGTGGTGGTGCTTTTTATTATCAGCTTTAGC	Reverse	XhoI	6xHis	AA 20-350, 60-350	pDJSVT84, pDJSVT85
prDJSVT215	GAGATATACCATGGGAATGAAAAAGACAGCTATCGCG	Forward	NcoI		OmpA 1-27 vector	pDJSVT86
prDJSVT216	GATCCTCGAGGGTACCCGCGGCCGCGTGGTGGTGGTGGTGGTGGGTGTTATCTTTCGGAG	Reverse	NotI, KpnI, XhoI	6xHis	OmpA 1-27 vector	pDJSVT86
prDJSVT259	GCATCGAATTCGAGATGTTGCTAAAATTTTAATAG	Forward	EcoRI		KO vector for strain 23726 Δ*fplA*	pDJSVT100
prDJSVT260	CGTAGCACTAGTCTAGAAAACTTTGAAAGTACAC	Reverse	SpeI		KO vector for strain 23726 Δ*fplA*	pDJSVT100
prDJSVT295	CATTTTTAGCAGATTATGAAAGTG	Reverse			*catP* primer to confirm pDJSVT100 and Δ*fplA*	Strain DJSVT01
U8F	AGAGTTTGATYMTGGCTCAG	Forward			16s rRNA for *F. nucleatum* verification	*Fusobacterium* strains
U1510R	GGTTACCTTGTTACGACTT	Reverse			16s rRNA for *F. nucleatum* verification	*Fusobacterium* strains
